# Single cell analysis identified IFN signaling activation contributes to the pathogenesis of pediatric *steroid-sensitive* nephrotic syndrome

**DOI:** 10.1186/s40364-025-00790-2

**Published:** 2025-05-24

**Authors:** Qiu-Yu Li, Fei Liu, Xiaoyi Li, Minchao Kang, Linnan Bai, Tong Tong, Chen Zheng, Yanyan Jin, Xiaojing Zhang, Yi Xie, Dandan Tian, Yuanqing Pan, Jingjing Wang, Haidong Fu, Na Jiao, Junnan Wu, JianHua Mao

**Affiliations:** 1 Department of Nephrology, The Children’s Hospital, Zhejiang University School of Medicine, National Clinical Research Center for Child Health, No. 3333, Binsheng Road, Binjiang District, Hangzhou, Zhejiang 310052 China; 2 Department of Nephrology, Zhejiang University Medical College Affiliated Sir Run Run Shaw Hospital, Qingchun Road 3rd, Hangzhou, Zhejiang Province 310016 China; 3 Zhejiang University School of Medicine, Zhejiang University, 866 Yuhangtang Road, Hangzhou, Zhejiang China; 4 Department of Clinical Laboratory, Children’s Hospital, Zhejiang University School of Medicine, National Clinical Research Center for Child Health, Hangzhou, China; 5 Department of Nephrology, Quanzhou Women’s and Children’s Hospital, Quanzhou, Fujian Province 362000 China; 6 State Key Laboratory of Genetic Engineering, Fudan Microbiome Center, School of Life Sciences, Fudan University, Shanghai, 200433 China

**Keywords:** Idiopathic nephrotic syndrome, scRNA-seq, IFN, PBMCs

## Abstract

**Background:**

Idiopathic nephrotic syndrome (INS) is a prevalent condition whose recurrence leads to multiple adverse effects. Previous studies on INS pathogenesis primarily focused on immune dysregulation, particularly T-cell changes and their correlation with cytokine shifts. Accumulating evidence suggests that B-cell dysfunction also plays a role. Nevertheless, a comprehensive understanding of the mechanisms and effective treatment strategies remains incomplete.

**Methods:**

This study investigates changes in gene expressions and cellular interactions of immune cells at a single-cell level using peripheral blood mononuclear cells (PBMCs). And subsequently validated through quantitative PCR (qPCR), enzyme-linked immunosorbent assay (ELISA), and flow cytometry.

**Results:**

We identified seven main clusters using unsupervised clustering of 103,213 high-quality single cells. Through unsupervised clustering, patient-specific T cells (IFI44L + CD4 + T cells) that exhibited a pronounced elevation of interferon-stimulated genes (ISGs) is identified. Activation of ISGs and interferon (IFN)-related pathways are also observed in other clusters. Specifically, this study demonstrates that interferon-γ (IFN-γ) plays a crucial role by promoting the interaction between B-cell activating factor (BAFF) and receptors on B cells. This interaction triggers the release of autoantibodies, thereby initiating INS pathogenesis. Furthermore, telitacicept has shown efficacy in treating pediatric patients with frequent relapse NS(FRNS).

**Conclusions:**

Overall, our findings underscore the role of interferon and its related pathways in INS pathogenesis, providing novel therapeutic interventions for NS.

**Supplementary Information:**

The online version contains supplementary material available at 10.1186/s40364-025-00790-2.

## Background

Nephrotic syndrome (NS) is the most common glomerular disease in childhood, with a morbidity spectrum spanning from 1.15 to 16.9 per 100,000 individuals [1]. Idiopathic nephrotic syndrome(INS), encompasses the majority (90%) of cases. Despite the typically favorable responses observed in most pediatric patients following a four-week steroid therapy regimen, these responses tend to be partial. The mechanism underlying INS has not been elucidated, characterized by dysregulated adaptive and innate immune signaling that drives glomerular permeability and proteinuria.

Previous studies indicated the alterations in T cells throughout the disease course. Specifically, T helper 17 (Th17) cells increased in adult new-onset INS patients, coupled with a decrease in regulatory T cells(Tregs) during relapse [2]. Interleukin(IL)−17, generated by Th17 cells, is implicated in glomerulosclerosis in focal segmental glomerulosclerosis(FSGS) [3]. Moreover, elevated levels of TNF-α and IL-1 have been detected in INS patients, favoring T-cell activation [4]. However, the pathogenicity of T cells alone fails to elucidate the pathogenesis of INS comprehensively. Treatments exclusively targeting T cells have demonstrated inconsistent efficacy. Recent findings have highlighted the involvement of B cells in INS pathogenesis. Alongside alterations in B lymphocyte counts [5], several autoantibodies produced by B cells have been identified, including anti-annexin A2 antibodies [6] and anti-nephrin antibodies [7]. Moreover, a correlation has been established between the levels of autoantibody and proteinuria [7]. Besides, cytokines may contribute to the pathogenesis of INS. A significant elevation of IL-2, IL-4 and interferon-γ(IFN-γ) was observed during relapse [8]. Markowitz GS et al. reported that 11 patients developed NS after IFN therapy but improved after discontinuation of IFN therapy [9]. Furthermore, several reported cases have highlighted the importance of IFN inducers in INS [10, 11]. Previous works have established that IFN-a drives B cell differentiation and enhanced antibody production in systemic lupus erythematosus [12]. However, the precise molecular mechanisms regulating autoantibody secretion in nephrotic syndrome (NS) and the role of interferon (IFN) signaling in orchestrating humoral autoimmunity remain elusive. We therefore postulate that IFN-mediated pathways constitute a critical driver of pathogenic autoantibody production in NS pathogenesis.

In the present study, we employed scRNA-seq of PBMCs from individuals with new-onset NS to investigate the mechanistic links between IFN activation, antibody production and NS pathogenesis to identify potential therapeutic targets. Initially, we first mapped the cell lineage and demonstrated the activation of the IFN response. Subsequently, intercellular communications analysis revealed elevated levels of B-cell activating factor(BAFF) and its receptors transmembrane activator and cyclophilin ligand interactor(TACI) and B-cell maturation antigen(BCMA), triggered by IFN. We also conducted quantitative polymerase chain reaction(qPCR), enzyme-linked immunosorbent assay(ELISA), and flow cytometry analyses to confirm the activation of BAFF and its receptors and the specific type of IFN in clinical cohorts. Overall, our research highlights the activation of IFN signaling pathways in INS pathogenesis.

## Materials and methods

### Study design

This study employed a prospective cohort design. We performed single-cell RNA sequencing of PBMC from five pediatric patients with new-onset steroid-sensitive INS patients, both before steroid treatment and within three days following the day of complete remission. Validation was subsequently conducted in an independent cohort through qPCR, ELISA, and flow cytometry. Additionally, an extra publicly available INS cohort was included for further validation.

### Data sources and human participants

This study was approved by the Ethics Committee of the Children’s Hospital, Zhejiang University School of Medicine (Number 2023-IRB-0024-P-01, 2024-IRB-0057-P-01). All donors aged 1–18 were recruited from the Children’s Hospital, Zhejiang University School of Medicine, between Mar 2023 and Jan 2024. Informed consent was obtained from the parents/guardians of the children.

Patients who firstly meet the INS criteria, defined as nephrotic-range proteinuria (urinary protein-to-creatinine ratio (uPCR) ≥ 2 mg/mg in first-morning void (≥ 200 mg/mmol), 24-h urine protein ≥ 50 mg/kg/day (1000 mg/m^2^/day), or ≥ 3 + by urine dipstick on three consecutive days), and hypoalbuminemia (serum albumin < 25 g/L) without known etiology [13] were recruited. The patients received 2 mg/kg prednisolone. Patients who achieve complete remission (uPCR ≤ 0.2 g/g, negative or trace dipstick test results) within four weeks following prednisolone treatment were enrolled. Additional inclusion criteria included an eGFR ≥ 90 ml/min/1.73m^2^. The main exclusion criteria were secondary causes, coexisting kidney or autoimmune disease, monogenic genetic disease, immunodeficiency, or active infections (detailed inclusion and exclusion criteria in Table S1). Healthy volunteers in the validation cohorts were recruited from the physical examination center. The main inclusion and exclusion criteria are as follows: aged 1–18 years, normal renal function, absence of proteinuria, no history of autoimmune or kidney disease, no recent infections. Raw scRNA-seq datasets from five healthy age-matched individuals obtained from the Gene Expression Omnibus (GEO) database (GSE214865) served as the control group in scRNAseq analysis. Additionally, raw scRNA-seq data from GSE233277 were used for complementary analysis.

### PBMCs isolation

A total of 2 ml of whole blood was collected in EDTA vacutainers. PBMCs were isolated by the Histopaque-1077 procedure within 2 h of venipuncture. Peripheral blood was diluted on ice with 0.9% saline (peripheral blood: 0.9% saline = 1:1). Subsequently, 2 ml of diluted blood was pipetted onto 2 ml of Ficoll (density gradient medium) and then centrifuged at 700 × g for 30 min at 20 °C. The cells were washed twice with 5 mL of 0.9% saline and centrifuged at 500 × g for 20 min at 20 °C. Subsequently, erythrocytes were lysed with 1 ml of 1 × red blood cell lysis buffer (MACS 130–094–183, 10 ×). Eventually, the cell pellet was resuspended in 1640 (containing 5% FBS) medium (Gibco, 11,835,030). For each sample, the cell viability rate was required to be more than 85%, as assessed by trypan blue staining, and the cell concentration was needed to fall within the range of 700–1200 cells/μl, as determined using a Countess II Automated Cell Counter.

### Single-cell sequencing and Chromium 10 × Genomics library preparation

The dissociated cell suspensions were loaded onto a 10 × Chromium instrument. After cell capture (targeting 5000 cells), cDNA amplification and library construction were carried out according to the manufacturer’s instructions (10X Genomics Chromium Single-Cell 3’ kit, V3). The resulting barcoded sequencing libraries were sequenced in 150 bp paired-end mode on a NovaSeq 6000 platform, with a target depth of 100 × and 20,000 reads per cell (performed by LC-Bio Technology Co., Ltd., Hangzhou, China).

### Cell filtering and quality control

The sequencing data were initially converted into FASTQ files using Illumina bcl2fastq software (version 2.20). Reference genome alignment was conducted by CellRanger (version 3.1.0) [14]. Sample demultiplexing was performed, the sequences were standardized to the same sequencing depth (normalization = mapped), and the gene–barcode matrices were subsequently recomputed. The resulting gene expression matrix file for each individual was imported into the Seurat R package (version 4.3.0) for subsequent analysis [15]. For quality control filtering, cells were retained based on the following criteria: 1) the number of expressed genes per cell was between 500 and 6,000; 2) the UMI counts were greater than or equal to 200; 3) the mitochondrial gene expression ratio was less than 20%; and 4) the average expression level of blood cell markers (*HBD*, *HBE1*, *HBM*, *HBA1*, *HBA2*, *HBQ1*, *HBB*, *HBZ*, *HBG1*, and *HBG2*) was less than 5% (Fig. S1C).

### Cell clustering and cell type subclustering

Preprocessed data were subsequently subjected to normalization and scaling. The individual datasets were merged through Seurat, followed by batch effect correction based on Harmony(version 0.1.0) [16]. Principal component analysis confirmed a reduction in batch effects following correction(Fig. S1A) Then, log transformation was conducted, regressing the percentage of genes related to mitochondria, hemoglobulin, and the cell cycle. The top 32 principal components were selected to reduce the dimensionality of the data. The shared nearest neighbor (SNN) module optimized clustering algorithm was employed through the “FindNeighbors” function. Then, doublets were detected and removed through *DoubletFinder* [17]. Clusters were visualized via uniform manifold approximation and projection (UMAP) and t-distributed stochastic neighbor embedding (t-SNE). Cell types were initially identified at a low resolution and were broadly categorized as T cells, B cells, myeloid cells, natural killer (NK) cells, plasmacytoid dendritic cells (pDCs), proliferative cells or plasma cells. Subsequent subanalyses using principal component analysis with a higher resolution were performed based on specific variable genes of each subcluster. The resulting data were visualized with Seurat, including heatmaps, dot plots, and violin plots.

### Marker gene and differentially expressed gene (DEG) analysis

Differential expression analysis between distinct cell clusters and different groups was conducted using the *FindAllMarkers* function in Seurat. Genes with a log fold change set to 0.5 and a significant cutoff false discovery rate (FDR) set to 0.05 were identified as marker genes. Cell clusters were annotated using well-known markers of immune cells [18]. To determine the specific DEGs that were involved in pathogenesis and the subsequent rescue by steroids, we compared the gene expression patterns of different cell types among steroid-responsive patients before (STS Pre) and after (STS Post) steroid treatment, and between the STS Pre and control (CT) samples, also with thresholds of FDR < 0.05 and log2fc > 0.5. These DEGs were further searched for overlapping genes(Table S3, S4). The overlapping DEGs (*IFI6*, *ISG15*, *MX1*, and *CXCR4*) were subjected to further validation through qPCR.

### Pathway enrichment analysis

We performed enrichment analysis with VISION (version 3.0.1). The gene sets used for pathway enrichment included Hallmark gene sets and Gene Ontology (GO) terms, which are publicly accessible in the Molecular Signatures Database (version 2023.1,https://www.gsea-MsigDB.org/gsea/MsigDB) (Table S5, S6).

### Trajectory analysis

We employed Monocle (version 2.26.0) [19] to reconstruct the lineage relationships and the transcriptional timeline of CD4 + T cells. Through the expression matrices of individual cells, these cells were assigned along pseudotemporal ordering paths.

### Interferon-stimulated genes (ISGs) score analysis

Interferon-stimulated genes (ISGs)(Table S7), which were previously defined as members of IFN-related modules, were used to determine the IFN activity score in each group (STS Pre, STS Post, and CT) and each cell cluster [20]. We utilized the *AddModuleScore* function in Seurat to calculate and assign an IFN response score for each cell based on a given list, and the scores were visualized in violin plots and box plots.

### Cell–cell communication analysis

CellChat (version 1.6.1) was used to explore intracellular communication. The interaction strengths between the two cell types were determined based on the average expression levels of specific ligand–receptor pairs in the two cell types [15]. Normalized data matrices from the STS Pre, STS Post and CT samples (healthy control (HC) and NS) served as inputs. To visualize disparities in total interaction scores among subclusters, we computed the ratio of interaction scores between different groups.

### Regulon analysis

Regulon analysis of relevant cell types was executed utilizing single-cell regulatory network inference and clustering (SCENIC) in Python (pySCENIC) (version 0.12.1) based on the underlying gene regulatory networks (GRNs) [21]. Specific transcription factors (TFs) were predicted based on the co-expression patterns within the gene expression matrix. TF-motif enrichment analysis employing RcisTarget was subsequently conducted. Co-expression modules associated with the TF that displayed significant motif enrichment were identified for further investigation. Indirect targets lacking motif support were eliminated, and each regulon included the TF and its potential direct-binding targets. Subsequently, we computed the activity of each regulon for each cell type using AUCell to assess the regulon enrichment. Differential regulon activities between the two groups were determined via a t-test, with a significance threshold of *P* < 0.05(Table S8).

### Flow cytometry and cell sorting

Flow cytometry was performed to determine the expression levels of TACI and BCMA on the surface of B-cell subsets with a FACSLyric ™ flow cytometer (BD) (at least 100,000 cells/sample). Flow cytometry data were analyzed with FlowJo software (v10.9.0). PBMCs were isolated from 10 NS patients and 12 healthy donors(Table 1). PBMCs were incubated with an appropriate combination of 7-amino-actinomycin D (7AAD), allophycocyanin (APC)-Cy7-conjugated anti-human CD45 (Cat. 561863a; BD Pharmingen), BV510-conjugated anti-human CD19 (Cat. 562,947; BD Pharmingen), PE-conjugated anti-human CD27 (Cat. 356,405; BioLegend), FITC-conjugated anti-human IgD (Cat. 555,778; BD Pharmingen), BV421-conjugated anti-human CD138 (Cat. 562,935; BD Pharmingen), Alexa Fluor (AF) 647-conjugated anti-human CD269 (BCMA, Cat. 357,517; BioLegend), and PE/Cyanine (PE/Cy)7-conjugated anti-human CD267 (TACI, Cat. 311,908; BioLegend) antibodies. Damaged and dead cells labeled with 7AAD were excluded from the analysis. The appropriate isotype and fluorescence minus one (FMO) control were used to adjust for background fluorescence and perform gating, and the results are reported as the percentage (%) of expression and the median fluorescence intensity (MFI) for naïve B cells (CD19 + IgD + CD27-), unswitched memory B cells (CD19 + IgD + CD27 +), switched memory B cells (CD19 + IgD-CD27 +), and plasma cells (CD27 + CD138 +). The gating strategy is shown in Fig. 6E and Fig. S5F.

**Table 1:** Basic characteristics of patients and healthy volunteers enrolled in validation experiments

	INS	Healthy	Statistics	*P* value
**qPCR cohort (n)**	n = 12	n = 12		
Age, M(P_25_-P_75_)	3.833 (3.271–7.438)	6.125 (4.458–8.354)	*Z* = −1.065	0.285
Male, % (n)	75.0 (9)	58.3 (7)		0.667^*^
**Flow cytometry cohort (n)**	n = 10	n = 12		
Age, M(P_25_-P_75_)	4.208 (2.563–8.042)	4.583 (3.188–8.938)	*Z* = −0.495	0.621
Male, % (n)	80.0 (8)	66.7 (8)		0.646^*^
**ELISA cohort (n)**	n = 20	n = 25		
Age, M(P_25_-P_75_)	4.21 (2.94–9.48)	6.00 (3.00–7.00)	*Z* = −0.252	0.801
Male, % (n)	55.0 (11)	52.0 (13)	*χ*^*2*^ = 0.040	0.841

*Abbreviations*: *qPCR* quantitative polymerase chain reaction, *INS* Idiopathic nephrotic syndrome, *ELISA* Enzyme Linked Immunosorbent Assay

^*^p-value for Fisher’s exact test

### Quantitative PCR (qPCR)

RNA was extracted from the PBMCs of 12 NS patients and 12 healthy children. Total RNA was extracted and purified by Direct-Zol ^TM^RNA Microprep (Zymo, USA). The purified RNA was reverse transcribed into cDNA through HiScript IV RT SuperMix for qPCR (+ gDNA wiper, Vazyme Medical Technology, Nanjing, CHN). The mRNA levels were analyzed by quantitative polymerase chain reaction (qPCR) through the CFX Connect Real-Time PCR Detection System (Bio-Rad). Each PCR was carried out in a 10 μL reaction mixture composed of 1 μL of cDNA template, 5 μL of PerfectStart Green qPCR SuperMix (TransGen Biotech, BJ, China), 0.5 mM forward primer, and 0.5 mM reverse primer. The primers used for qPCR were as follows: human IFI6: forward 5′- TGCTTCTCTTCTCTCCTCCAA-3′, reverse 5′- GCTCTCCGAGCACTTTTTCTT-3′; human ISG15: forward 5′-CGCAGATCACCCAGAAGATCG-3′, reverse 5′-TTCGTCGCATTTGTCCACCA-3′; human MX1: forward 5′- GTTTCCGAAGTGGACATCGCA-3′, reverse 5′- CTGCACAGGTTGTTCTCAGC-3; human CXCR4: forward 5′- ACTACACCGAGGAAATGGGCT-3′, reverse 5′-CCCACAATGCCAGTTAAGAAGA-3; U6 was used as the internal control. The relative mRNA expression levels were calculated via the 2-ΔΔCt method.

### Enzyme-linked immunosorbent assay (ELISA)

Serum was collected from 20 NS patients and 25 healthy volunteers upon admission to the hospital and then stored at −80 °C. The serum expression levels of BAFF (human BAFF ELISA; Proteintech, United States; sensitivity = 0.4 pg/mL), IFN-α (human IFNA1 ELISA; Proteintech, United States; sensitivity = 1.5 pg/mL), IFN-γ (human IFN-gamma ELISA Kit; Proteintech, United States; sensitivity = 0.4 pg/mL), and IFN-λ (AuthentiKine™ Human IL-29 ELISA Kit; Proteintech, United States; sensitivity = 3.3 pg/mL) were measured through ELISA according to the manufacturer’s protocols.

### Statistical analysis

All statistical analyses were conducted using R (version 4.2.1), Prism software (version 9.3.1) and SPSS 27.0. The data distribution was assessed, and comparisons were conducted based on the normality of the data. Students' *t-tests* (paired or unpaired) or the nonparametric Wilcoxon signed-sum tests (paired or unpaired) were used for two-group comparisons, while one-way ANOVA was used for multiple groups. Categorical data were tested using the chi-square test or Fisher’s exact test. ROC curve analysis was conducted with Prism software to analyze the gene expression in each cell type. Statistical significance is denoted as *P* < 0.05 (*),* P* < 0.01 (**), *P* < 0.001 (***), or *P* < 0.0001 (****).

## Results

### Study participants

**Fig. 1:**
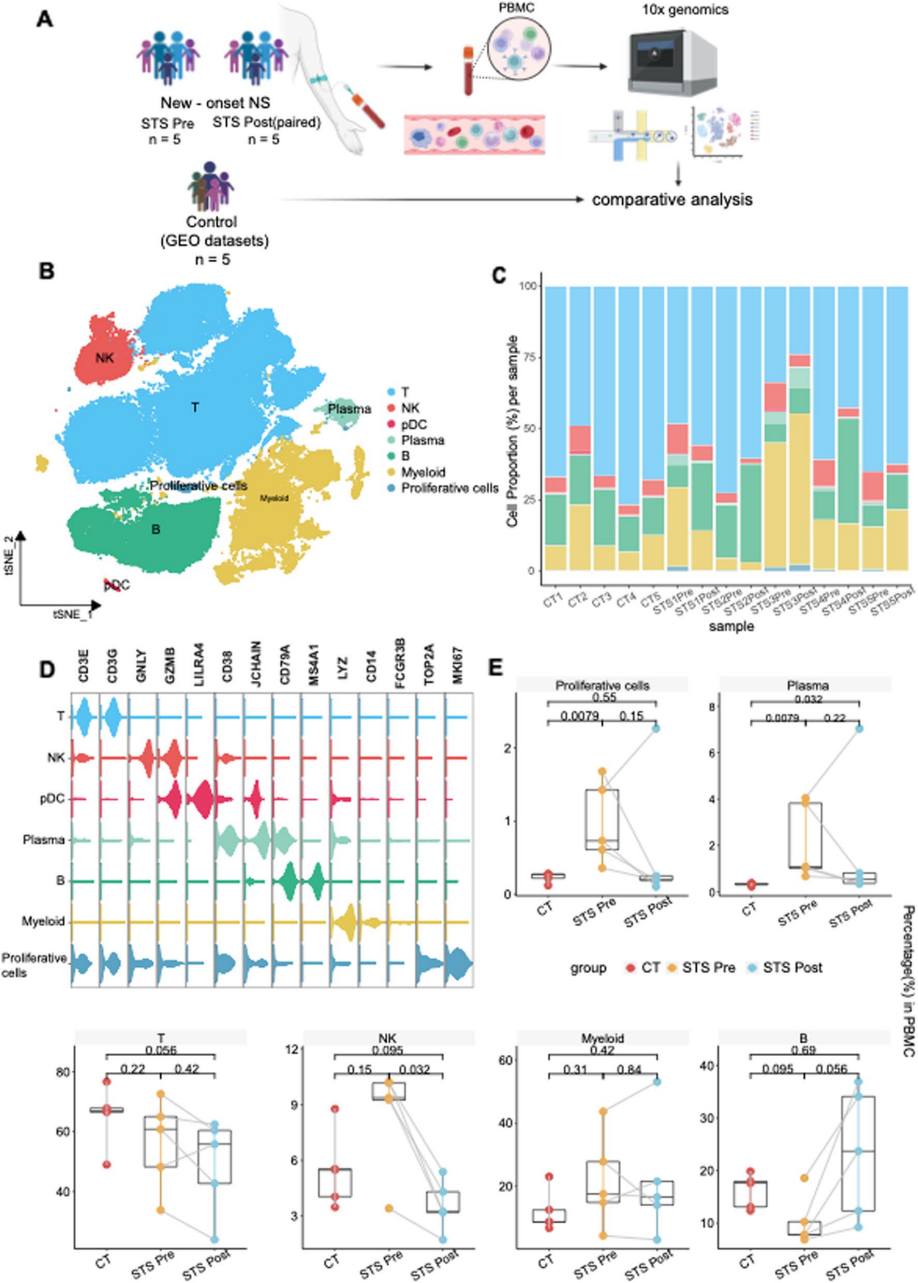
Single-cell transcriptional profiling of PBMCs from NS patients. **A** Flowchart overview of PBMCs collection followed by scRNA-seq experiments. n = 5 steroid-responsive NS patients before (STS Pre) and after (STS Post) steroid treatment. The control group (CT) was obtained from the GEO(GSE214865) (n = 5). **B** t-SNE projections of PBMCs from all enrolled individuals. **C** Bar chart presenting the relative proportions of each cluster (T cell, B cell, DC, Plasma cell, NK cell, myeloid cell, proliferative cell) in samples derived from scRNA-seq in PBMCs. **D** Violin plot presenting the expression levels of markers across each cluster. The violin size represents the expression level of the marker of interest. **E** Boxplot comparing the proportions of each cell type across the groups. The STS Pre vs. CT and STS Post vs. CT sample comparisons show exact *P* values determined by the Wilcoxon rank-sum test. Pre- vs. post-STS scores were calculated via the paired two-sample Wilcoxon signed-sum test

To gain a comprehensive insight into the immune landscape and the underlying mechanism of INS, we collected PBMCs from 5 new-onset INS patients who were sensitive to steroids before(STS Pre) prednisolone treatment and after complete remission(STS Post) for 10X Genomics scRNA-seq. Additionally, raw scRNA-seq data from five paired healthy children were collected from GEO(GSE214865) for comparative analysis(CT) (Fig. 1A). The average age of the INS patients was 4.9 years, comparable to the control group's 4.8 years(detailed data in Fig. S1F).

Subsequently, 42 patients with new-onset NS and 49 healthy volunteers were recruited for the validations. The detailed baseline characteristics are summarized in Table S2. To mitigate bias, no significant demographic differences were found between the two groups in each validation(qPCR, ELISA, flow cytometry) (Table 1).

Additionally, GSE233277, including samples from 4 INS patients with active disease and 4 healthy controls, aged between 7 and 18 years old, were used for complementary analysis (Fig. S7).

### Proliferation of multiple lymphocytes in the pretreatment group

After filtering, 103,213 high-quality single cells were retained for further analysis. These cells were divided into seven distinct clusters: T cells(*CD3G*, *CD3E*), B cells(*MS4A1*, *CD79A*), plasma cells(*CD38*, *JCHAIN*), natural killer(NK) cells(*GNLY*, *GZMB*), plasmacytoid dendritic cells(pDCs)(*LIRA4*, *JCHAIN*), other myeloid cells(*CD14*, *LYZ*) and proliferative cells(*TOP2A*, *MKI67*)(Fig. 1B-D, Fig. S1A-1D). A significant increase in plasma cells was evident in the STS Pre group(*P* = 0.008), which decreased after treatment. Contrary to plasma cells, B cells were diminished before treatment, potentially indicating B cell maturation and antibody secretion in the pathogenesis of NS [22]. Another notable increase was observed in proliferative cells(*P* = 0.008), which comprised a mixture of NKT and plasma cells, indicating lymphocyte proliferation during active disease. This proliferation was not limited to plasma and proliferative cells, encompassing NK and myeloid cells, indicating cell expansion in INS (Fig. 1E, Fig. S1E). Collectively, these results demonstrate a proliferative state of lymphocytes and suggest an underlying immune disorder in INS.

### Patient-specific T cells (IFI44L + CD4 + T cells) are identified in INS pathogenesis

**Fig. 2:**
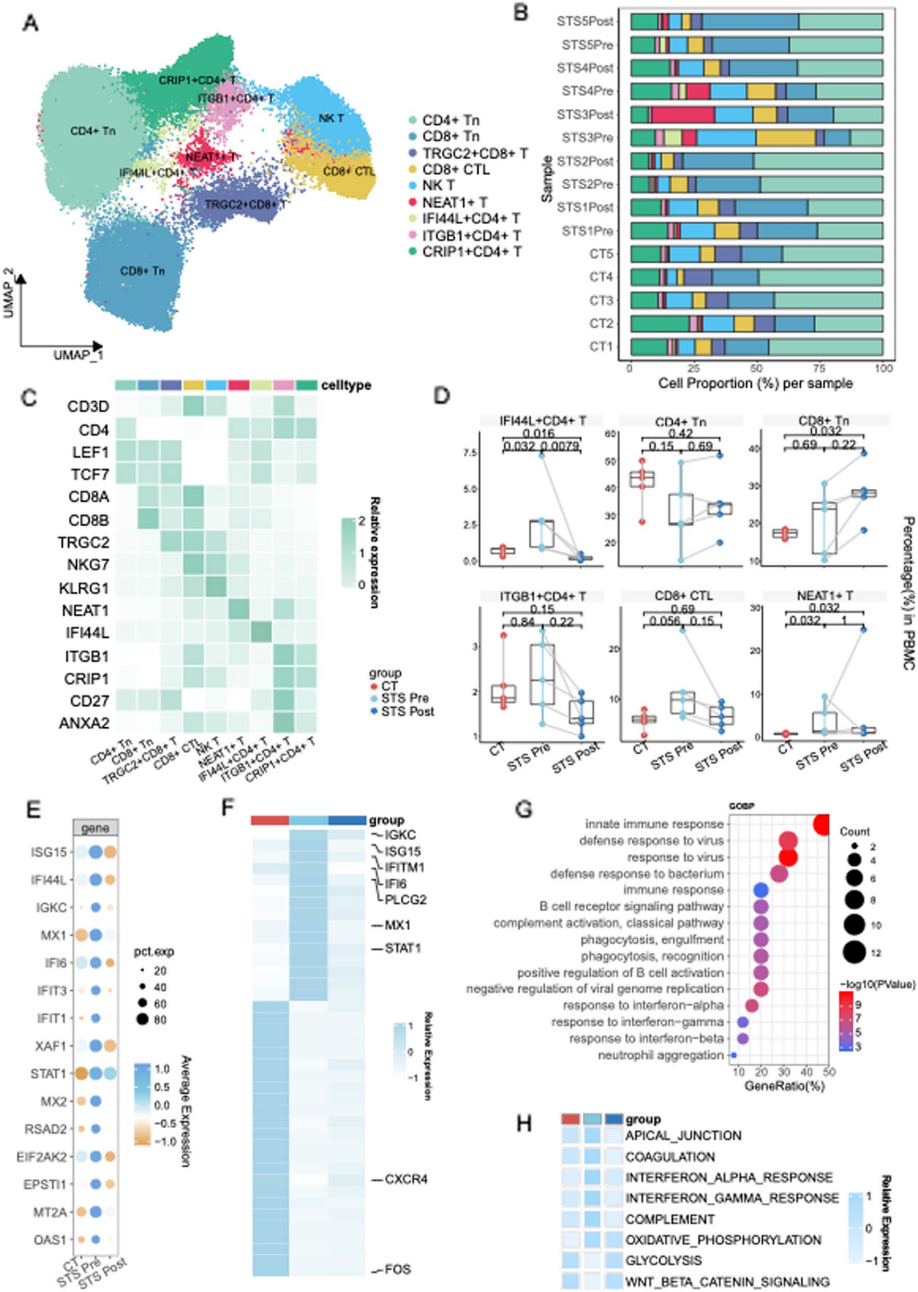
Proliferation in T subclusters exhibits high expression of IFN-related genes. **A** UMAP dimensionality reduction embedding of T lymphocytes from all profiled samples. **B** A bar chart represents each sample's relative proportions of T lymphocyte clusters. **C** Heatmap showing the expression levels of the markers across each cluster. The color intensity indicates the expression of the marker of interest. **D** Boxplot comparing the proportions of CD4 + naïve T cells, CD8 + naïve T cells, CD8 + CTLs, IFI44L + CD4 + T cells, ITGB1 + CD4 + T cells and NEAT + T cells across the groups. The STS Pre vs. CT and STS Post vs. CT sample comparisons show exact P values determined by the Wilcoxon rank-sum test. Pre- vs. post-STS scores were calculated via the paired two-sample Wilcoxon signed-sum test. **E** Dot plot showing the top 15 marker genes among IFI44L + CD4 + T cells across the groups. The dot size indicates the percentage of cells expressing the marker of interest. The color intensity represents the mean scaled expression of genes in the expressing cells. **F** Heatmap showing the DEGs among T cells across groups. The color scheme indicates the relative expression from − 1 (white) to 1 (dark blue). **G** Enriched pathways from Gene Ontology Biological Process Enrichment Analysis for IFI44L + CD4 + T cells. **H** Heatmap representing the enrichment of Hallmark gene sets in the MSigDB within T cells across groups

To delve deeper into the transcriptomic alterations in immune cells before and after steroid treatment, we conducted a subanalysis focusing on T-cell data. Thus, we discerned eight subclusters among T cells (Fig. 2A, C): CD4 + naïve T cells (CD4 + Tn) (*LEF1*, *CD4*), CD8 + naïve T cells (CD8 + Tn)(*LEF1*, *CD8A*, and *CD8B*), CD8 + cytotoxic T lymphocytes (CTLs) and NKTs(*NKG7* and *KLRG1*). Functional marker genes(*NEAT1*, *IGTB1*, *CRIP1*, *IFI44L*, and *TRGC2*) were used to define NEAT + T, IGTB1 + CD4 + T, CRIP1 + CD4 + T, IFI44L + CD4 + T, and TRGC2 + CD8 + T cells respectively.

Patients displayed a notable reduction in CD4 + Tn and TRGC2 + CD8 + T cells, while IGTB1 + CD4 + T cells, NKTs and CTLs increased before treatment, reverted to levels akin to those in the control group after treatment, indicating a proliferative capacity in T lymphocytes(Fig. 2B, D). TRGC2 + CD8 + T cells, enriched in the control group, showcased pathways related to cell cycle and mitochondrial metabolism, indicating robust remodeling(Fig. S2A-2C). IGTB1 + CD4 + T cells, characterized by high expression of *CD27*, *ANXA1*, and *ANXA2*, are presumed to be memory T cells. These elevated T-cell subclusters may suggest that the activated immune response induced functional T-cell proliferation during the disease.

Notably, IFI44L + CD4 + T cells, significantly augmented in the STS Pre group(Fig. 2D) (STS Pre vs CT, *P* = 0.032; STS Pre vs STS Post, *P* = 0.008), were identified as a patient-specific T subset. This subgroup exhibited a pronounced elevation in the expression of IFN-stimulated genes(ISGs)(*ISG15*, *STAT1*, etc.)(Fig. 2E). Enrichment analysis depicted B-cell activation and IFN-related responses(Fig. 2G), suggesting the activation of IFN-related pathways in the INS. Trajectory analysis revealed that these cells occupied an intermediate state between CD4 + Tn and IGTB1 + CD4 + T cells(memory T cells)(Fig. S2F). Additionally, NEAT + T cells significantly increased before treatment and were also enriched in response to IFN(Fig. S2D).

Given the correlation between IFI44L + CD4 + T cells and IFN-related pathways, we aimed further to delineate discrepancies in gene expression across groups. Specific expressed gene analysis of T cells revealed high expression of ISGs(*ISG15*, *MX1*, etc.) in the STS Pre group (Fig. 2F). Further enrichment analyses also revealed the IFN-α and IFN-γ responses before treatment, particularly evident in CD8 + CTLs, ITGB1 + CD4 + T, IFI44L + CD4 + T and NK T cells(Fig. 2H, Fig. S2E). These findings suggest that IFI44L + CD4 + T cells and IFN-related pathways may contribute to the pathogenesis of NS.

### B lymphocytes are activated and maturated during the pathogenesis of INS

**Fig. 3:**
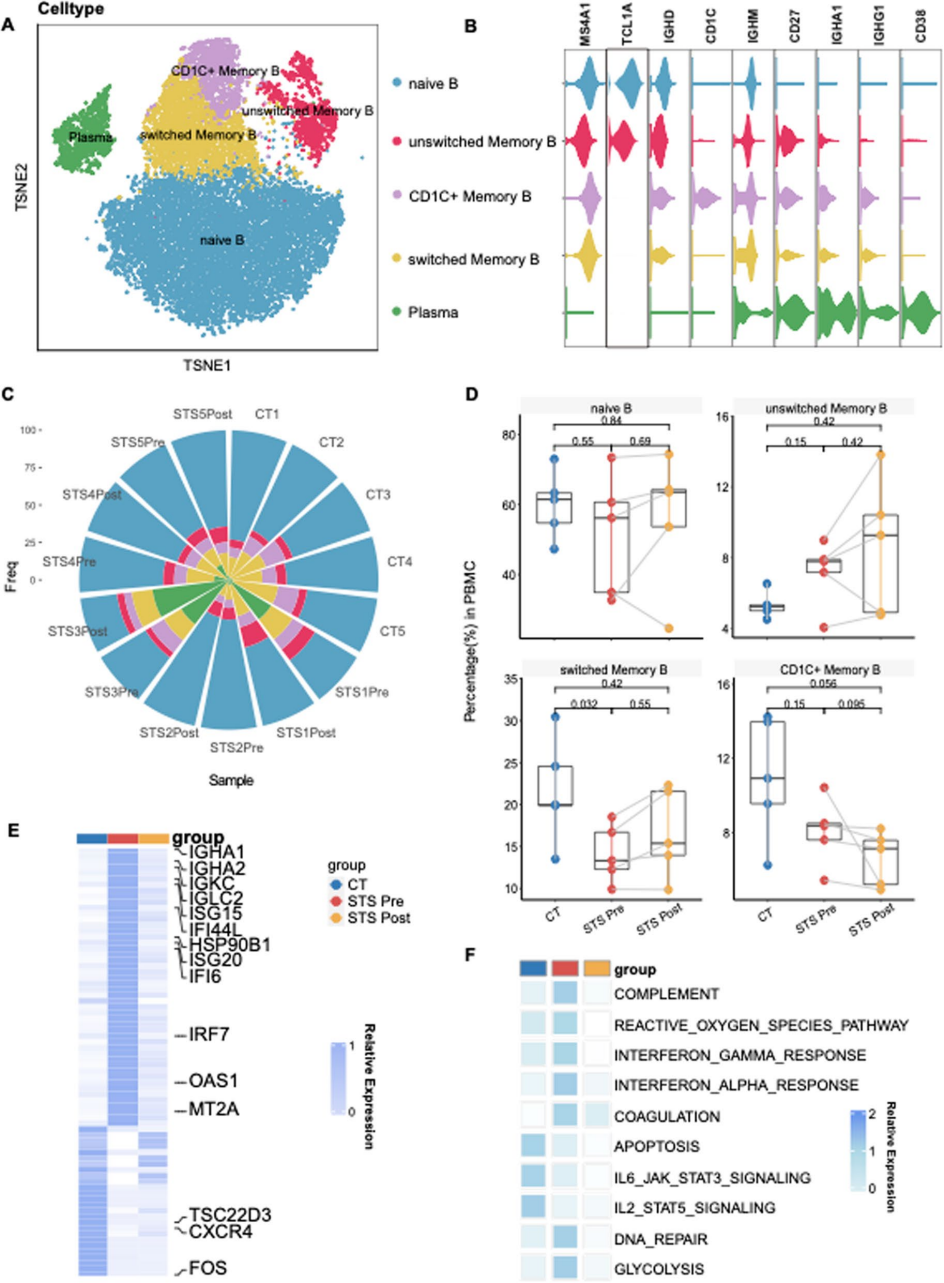
Dissection of B cells and plasma cells proposed evidence of B cell activation. **A** t-SNE projections of B lymphocytes from all profiled samples. **B** Violin plot showing the expression levels of markers across each cluster in B lymphocytes and plasma cells. The violin size represents the marker of interest. naïve B cells express *TCL1A* and *MS4A1*). Unswitched memory B cells exclusively exhibited high expression of *IGHD* and *IGHM*, whereas switched memory B cells displayed high *IGHA1* and *IGHG1.* CD1C + memory B cells highly expressed *CD1C.* **C** A circle bar chart representing the relative proportions of B lymphocytes in each cluster in the samples. **D** Boxplot comparing the proportion of switched memory B cells across the groups. The STS Pre vs. CT and STS Post vs. CT sample comparisons show exact P values determined by the Wilcoxon rank-sum test. Pre- vs. post-STS scores were calculated via the paired two-sample Wilcoxon signed-sum test. **E** Heatmap showing the DEGs within B lymphocytes across groups. The color scheme is based on the relative expression from 0 (white) to 1 (dark blue). **F** Heatmap represents the Hallmark gene set enrichment in the MSigDB within B cells across groups

Apart from T cells, B lymphocytes were implicated in INS pathogenesis. We identified four subclusters of B cells apart from plasma cells: naïve B cells (*TCL1A* and *MS4A1*) and three memory B cells(*CD27*)(Unswitched memory B cells, switched memory B cells, and CD1C + memory B cells) to clarify the underlying variations(Fig. 3A-B). Reductions in naïve B cells and switched memory B cells were apparent, whereas the percentage of plasma cells was significantly higher in the STS Pre group(Fig. 3C-D), indicating that disease progression, inflammation and autoantibody may contribute to the differentiation and maturation of B cells [22, 23].

Given the high expression of ISGs in T cells in the STS Pre group, we extended our DEG analysis to B cells. Notably, *IGHA1* and *IGHA2* exhibited elevated levels in the STS Pre group, indicating immunoglobulin switching. Additionally, ISGs(*ISG15*, *IFI44L*, etc.) were prominently upregulated in the STS Pre group(Fig. 3E), indicating enrichment in ΙFN-α and IFN-γ responses(Fig. 3F, Fig. S3A), particularly in switched memory B cells and plasma cells. This suggests a potential interaction between IFN-related pathways and B cell maturation.

### Myeloid cells and NK cells are involved in the IFN signaling

**Fig. 4:**
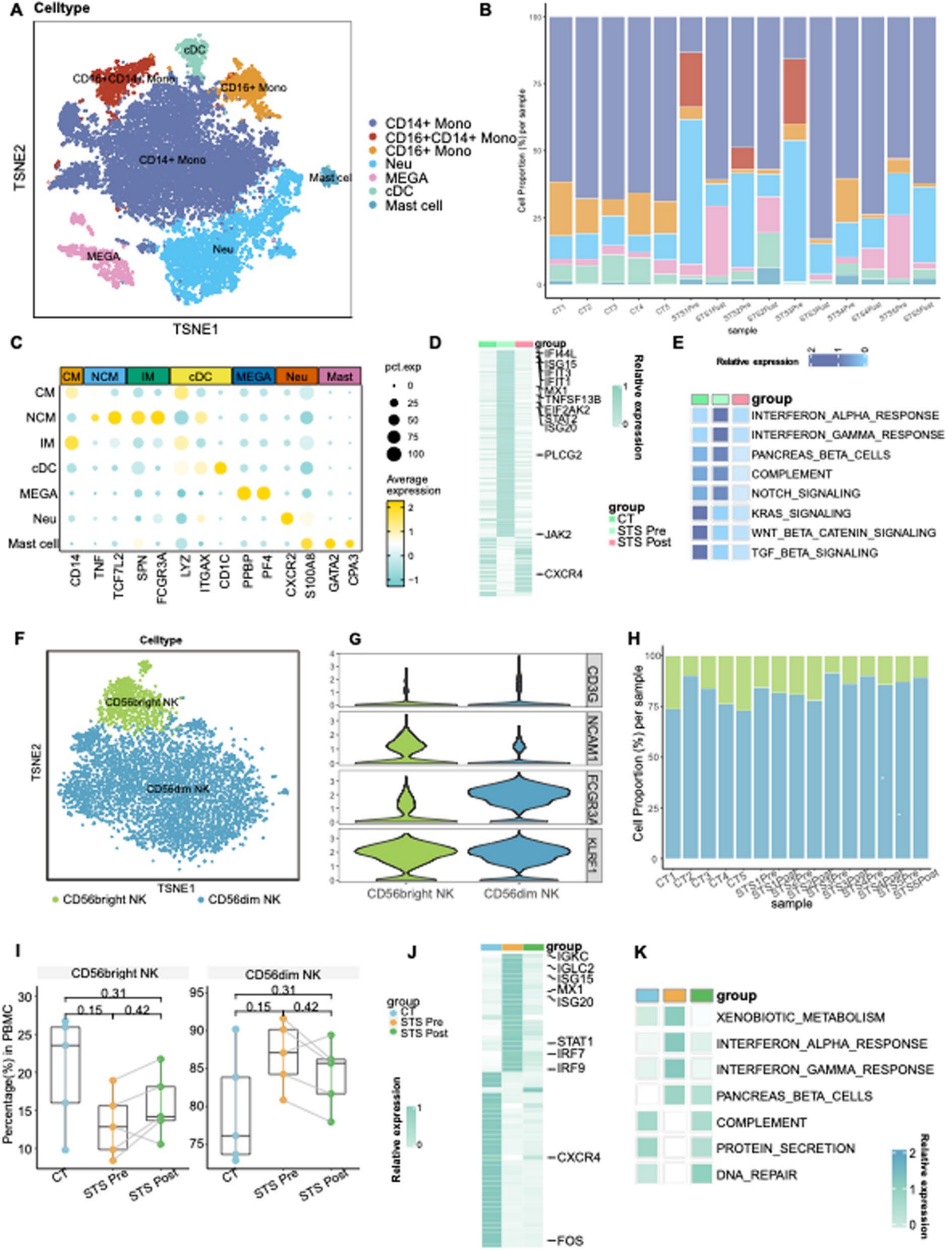
Myeloid cells and NK cells are involved in IFN response. **A** t-SNE projections of myeloid cells from all profiled samples. **B** A bar chart representing the relative proportions of myeloid cells in each cluster in the samples. **C** Dot plot showing marker genes in myeloid cells. conventional DCs (cDCs) by *LYZ* and *CD1C*. Mast cells were distinguished by *GATA2* and *CPA3.* Neutrophils were defined by *CXCR2* and *S100A8* expression, with megakaryocytes by *PPBP* and *PF4.* Monocytes were identified by high expression of *CD14* and *CD16*. Monocytes (*CD14*, *CD16*) were re-clustered into three subclusters. Classical monocytes (CMs) exhibited high *CD14* expression and low *CD16* expression, while nonclassical monocytes (NCMs) exhibited the opposite pattern. Intermediate monocytes (IMs), exhibiting features of both CMs and NCMs, expressed both *CD14* and *CD16.* The dot size indicates the percentage of cells expressing the marker of interest. The color intensity represents the mean scaled expression of genes in the expressing cells. **D** Heatmap showing the DEGs in myeloid cells across groups. **E** Heatmap represents the Hallmark gene sets enrichment in the MSigDB within myeloid cells across groups. **F** t-SNE projections of NK cells from all profiled samples. **G** violin plot showing the expression levels of markers across each cluster in NK cells. The violin size represents the marker of interest. CD56dim NK cells were identified with high *FCGR3A* expression and absence of *NCAM1*, while CD56bright NK cells exhibited a high level of *NCAM1* and a low level of *FCGR3A.* **H** The bar chart represents the relative proportions of NK cells in each cluster. **I** Boxplot comparing the proportion of each cluster among NK cells across the groups. Shown in the STS Pre vs. CT and STS Post vs. CT sample comparisons are exact P values determined by the Wilcoxon rank-sum test. Pre- vs. post-STS scores were calculated via the paired two-sample Wilcoxon signed-sum test. **J** Heatmap showing the DEGs in NK cells across groups. **K** Heatmap represents the Hallmark gene set enrichment in the MSigDB within NK cells across groups

Given the activation of the IFN response in both T and B lymphocytes, we also ascertain the responses to IFN in myeloid and NK cells. Monocytes were clustered into three subclusters(Classical monocytes(CMs), nonclassical monocytes(NCMs) and intermediate monocytes(IMs)(Fig. 4A, C). Inflammatory cytokine-secreting CMs and oxidative respiration-enriched NCMs exhibited a substantial reduction, whereas IM was significantly increased in the STS Pre group(Fig. 4B, Fig. S3B). IM is considered the principal effector of inflammation [24, 25]. Its accumulation may be correlated with disease progression [26–28]. The reduction of NCMs, which are enriched in oxidative respiration, may linked to their consumption to maintain homeostasis(Fig. S3C) [29, 30]. Furthermore, DEGs among three monocyte types also exhibited greater expression of numerous ISGs (Fig. 4D), along with the enrichment of IFN responses in the STS Pre group(Fig. 4E, Fig. S3D).

Apart from monocytes, conventional DCs(cDCs)(*LYZ*, *CD1C*) displayed a significant reduction in patients(Fig. 4C, Fig. S3B), suggesting a depletion in antigen presentation in NS. As IFN-I-producing cells [31], pDC increased slightly before treatment, followed by a marked decrease post-remission(Fig. S1E), suggesting the potential involvement of pDCs and IFN in NS pathogenesis. Despite the reduction of cDCs, both cDCs and pDCs exhibited an increased expression of ISGs(Fig. S2G, S4A) and were enriched in IFN responses in the STS Pre group(Fig. S2H, S4D). Likewise, the analysis of mast cells (*GATA2*, *CPA3*) also indicated IFN responses in the STS Pre group(Fig. S4B- S4C).

Apart from myeloid cells, CD56dim NK cells(absence of *NCAM1*), representing the majority of circulating NK cells, showed an increase in the STS Pre group, whereas CD56bright NK cells(a high level of *NCAM1*) decreased(Fig. 4F-I). The variation in the two NK subsets may indicate a cytokine-driven response during disease. DEGs and enrichment analysis among NK subclusters also highlighted the activation of the ΙFN in the STS Pre group, particularly in CD56 bright NK cells(Fig. 4J-K, Fig. S4E).

### The pathogenesis of NS is strongly correlated with IFN-stimulated genes

**Fig. 5:**
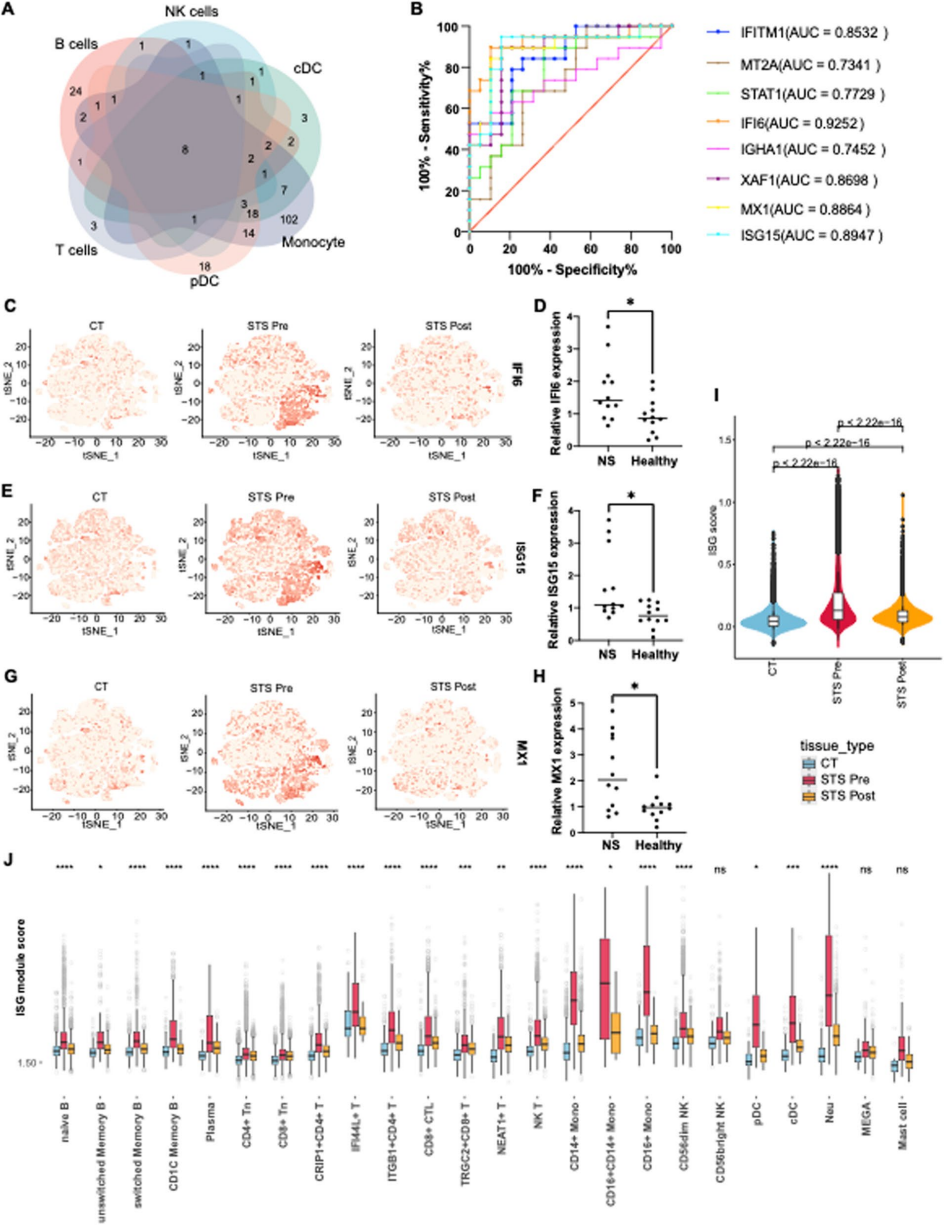
The activation of IFN-stimulated genes is widely observed across various cell types. **A** Venn plot of the overlapping genes highly expressed in the STS Pre group among B lymphocytes, T cells, monocytes, NK cells and neutrophils. **B** The receiver operating characteristic (ROC) curve and area under the curve (AUC) of overlapping genes highly expressed before treatment in all cell types. **C** T-SNE analysis of IFI6 expression in the three groups. **D** The relative expression of IFI6 mRNA across groups was determined via qPCR. **E** T-SNE analysis of ISG15 expression in the three groups. **F** The relative expression of ISG15 mRNA across groups was determined by qPCR. **G** T-SNE analysis of MX1 expression in the three groups. **H** The relative expression of MX1 mRNA across groups was determined through qPCR. **I** Violin plot of the ISGs across groups. Significance was evaluated with one-way ANOVA. **J** The score of ISGs among cell subtypes across groups. Significance was evaluated with one-way ANOVA. Statistical significance is denoted as *P* < 0.05 (*),* P* < 0.01 (**), *P* < 0.001 (***), or *P* < 0.0001 (****)

In light of the preliminary analysis, we have established an association between the IFN pathway and INS pathogenesis. Furthermore, the ISGs score was significantly higher among distinct cell types in the STS Pre group(Fig. 5I-J). Given the similarity of IFN-related genes across the various subclusters, we performed an intersection of all DEGs among B cells, T cells, pDCs, monocytes, cDCs and NK cells(Fig. 5A, Fig. S4F, Table S3-S4). This analysis revealed eight upregulated overlapping genes(*IFITM1*, *MT2A*, *STAT1*, *IFI6*, *XAF1*, *MX1*, *ISG15* and *IGHA1*)(Fig. 5B) and six downregulated genes(*FOSB*, *JUN*, *PPP1R15A*, *RPL13A*, *CXCR4*, and *TSC22D3*) in the STS Pre group(Fig. S4G). To confirm the activation of ISGs, PBMCs from 12 INS patients and 12 healthy donors(Table 1) were extracted to assess the relative expression of *IFI6*, *ISG15*, *MX1*, and *CXCR4*, which had the largest area under the curve(AUC) values(Fig. 5C, E, G, Fig. S4H) via qPCR. Accordingly, compared to healthy donors, the expression levels of *IFI6* (*P* = 0.016) (Fig. 5D), *ISG15* (*P* = 0.017) (Fig. 5F) and *MX1* (*P* = 0.024) (Fig. 5H) were significantly higher in patients, whereas the expression level of *CXCR4* (*P* = 0.002) (Fig. S4I) was significantly lower.

Moreover, transcriptional factors (TFs) analysis revealed the activation of interferon regulatory factor (*IRF*)7, signal transducer and activator of transcription (STAT)1 and STAT2 in the STS Pre group, corroborating the activation of the IFN-related pathway(Fig. S5A-5D, Table S8). These findings confirm that the IFN-related pathway is involved in the pathogenesis of INS.

### IFN-γ facilitates B lymphocyte maturation and antibody production

**Fig. 6:**
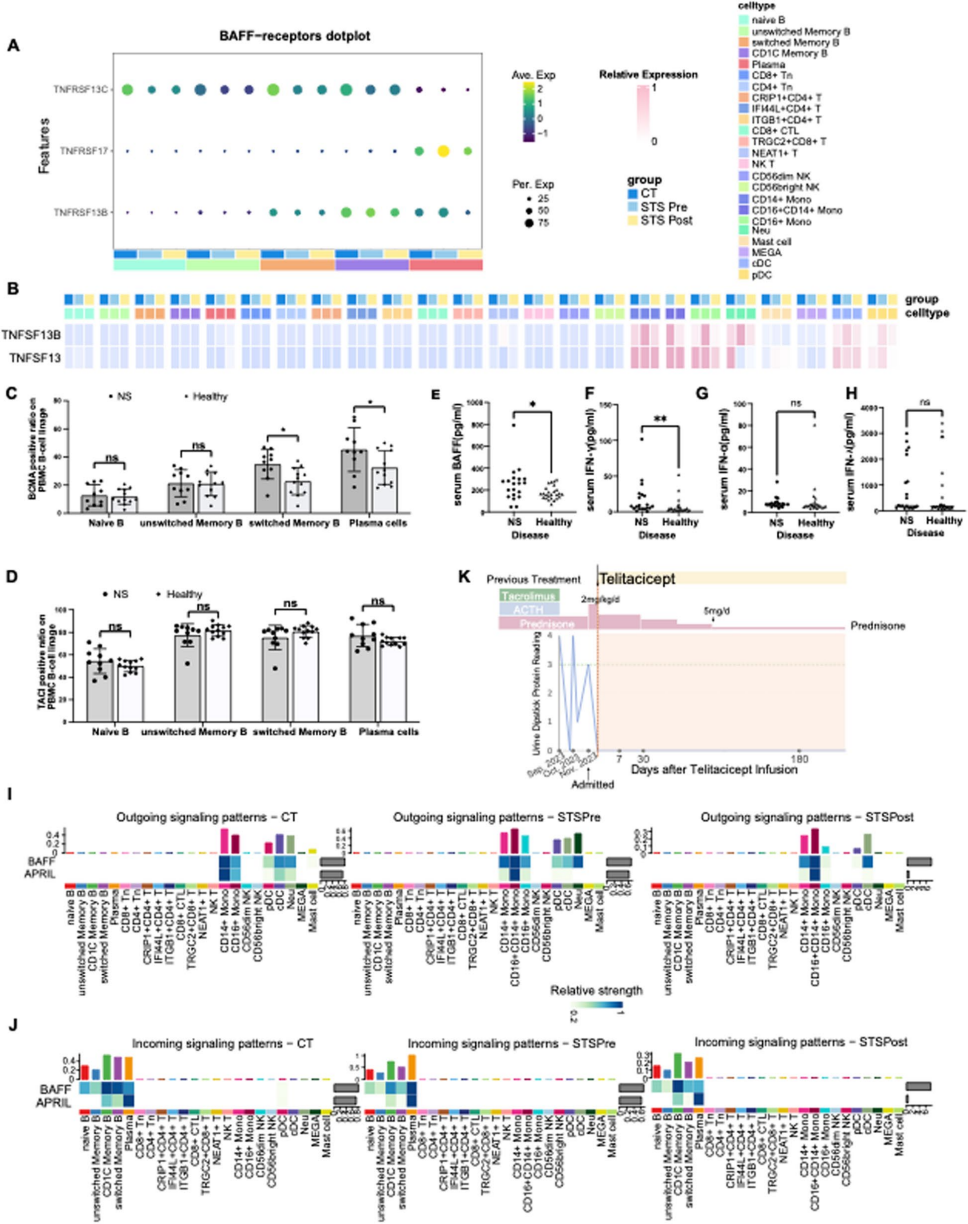
BAFF and its receptors are highly expressed during active disease, validated in clinical samples. **A** A dot plot showing the expression levels of the receptors (TNFRSF13C, TNFRSF17, and TNFRSF13B) of BAFF. The dot size indicates the percentage of cells expressing the marker of interest. The color intensity represents the mean scaled expression of genes in the expressing cells. **B** Heatmap showing the expression levels of TNFSF13B and TNFSF13 in each cell type across groups. **C** Surface expression data of BCMA on naïve B cells, unswitched memory B cells, switched memory B cells and plasma cells through flow cytometry. Samples were obtained from NS patients and healthy volunteers. The mean ± SD was used to express these data. ns, *p* > 0.05, *p < 0.05. **D** Surface expression data of TACI on naïve B cells, unswitched memory B cells, switched memory B cells and plasma cells through flow cytometry. Samples were obtained from NS patients and healthy volunteers. The mean ± SD was used to express these data. ns, *p* > 0.05, *p < 0.05. **E** The relative expression level of BAFF in the serum of NS patients compared to that of healthy individuals. *p < 0.05. **F** The relative expression level of IFN-γ in the serum of NS patients compared to that of healthy individuals. *P* < 0.01 (**).**G** The relative expression level of IFN-α in the serum of NS patients compared to that of healthy individuals. ns, *p* > 0.05. **H** The relative expression level of IFN-λ in the serum of NS patients compared to that of healthy individuals. ns, *p* > 0.05. **I**. Outgoing signaling patterns of BAFF and APRIL across groups. **J** Incoming signaling patterns of BAFF and APRIL across groups. **K** Change in the urine dipstick protein level of a patient with frequent relapse NS during the follow-up period

Though we have demonstrated the involvement of IFN-related pathways in the pathogenesis of NS, the specific role of activated IFN signaling remains elusive. Recently, several autoantibodies such as anti-Annexin A2 and anti-nephrin antibodies, have been detected in NS [7, 32, 33]. Therefore, we hypothesized that IFN may affect B-cell maturation and antibody production through activated ISGs in the pathogenesis of INS [34, 35]. B-cell maturation and differentiation depend on B-cell surface receptors. The BAFF receptor(BAFFR, TNFRSF13C) participates in B-cell maturation, while B-cell differentiation and antibody secretion are predominantly mediated by TACI(TNFRSF13B) and BCMA(TNFRSF17) [36]. Consistent with our hypothesis, scRNA-seq identified elevated expression of BCMA and TACI (Fig. 6A). Flow cytometry confirmed statistically significant BCMA upregulation in patients (45.23 ± 15.57 vs. 32.54 ± 12.06 AU in controls, *t* = 2.156, *p* = 0.043), with a slight increase of TACI (77.08 ± 9.82 vs. 77.05 ± 3.59 AU, *t* = 1.536, *p* = 0.153) (Fig. 6C-D). These receptor changes coincided with the overexpression of BAFF, a ligand generated by IRFs and regulated by IFN [37], in monocytes, neutrophils, and dendritic cells, as observed in our scRNA-seq data(Fig. 6B). Serum *BAFF* levels were 1.6-fold higher in patients (260.46 pg/mL(142.76–301.64) vs. 162.32 pg/mL(129.93–209.93); *Z* = −2.056, *p* = 0.040) (Fig. 6E). These findings are consistent with established *BAFF*-INS associations [38, 39]. In addition to BAFF, a proliferation-inducing ligand (APRIL, TNFSF13) has been suggested to be enriched in active INS [40]. However, our data didn’t show higher APRIL levels(Fig. 6B). Furthermore, cell–cell ligand-receptor interaction analysis also indicated the activation of the BAFF pathway rather than the APRIL pathway(Fig. 6I-J). Thus, these results collectively reveal that IFN activates ISGs, stimulating the expression of BAFF in monocytes, neutrophils, and dendritic cells. The highly expressed BAFF then binds to B-cell receptors (BCMA and TACI), subsequently stimulating B-cell maturation and antibody production(Fig. S8).

The IFN system comprises three types of interferon cytokines: type I (IFN-α and IFN-β, etc.), type II(IFN-γ), and type III IFNs(IFN-λ 1–4). To evaluate this resource, we performed ELISAs to test the serum levels of IFN-α, IFN-γ, and IFN-λ in 20 INS patients and 25 healthy volunteers(Table 1). IFN-γ levels were significantly elevated in patients (7.72(4.46–23.52) vs. 3.39(2.38–6.56) pg/mL; *Z* = −2.627, *p* = 0.009), whereas IFN-α (7.36 (6.01–8.85) vs. 6.06 (5.10–7.52); *Z* = −1.645, *p* = 0.100) and IFN-λ (196.50 (127.14–1920.01) vs. 172.76(118.24–774.33); *Z* = −0.662, *p* = 0.508) showed no statistically significant differences compared to healthy controls (Fig. 6F-H). These findings specifically implicate IFN-γ in INS pathogenesis. IFN-γ is mainly produced by T lymphocytes and NK cells [41]. CD56bright NK cells, predominantly responsible for generating IFN-γ in the initial host defense against infections [42], exhibit a reduction in the STS Pre group(Fig. 4I). Hence, T cells are recognized as the major source of sustained IFN-γ in the immune response(Fig. S5E) [42]. Consequently, we suggest that T cells primarily contribute to IFN-γ, with potential participation from NK cells as well.

### The activation of the IFN-related pathway is validated in an extra INS cohort

Recently, Al-Aubodah TA et al. [40] focused on B cells in the pathogenesis of INS in Canadian patients. TF analysis in this study suggested activation of the IFN response. However, no further analysis was performed. To further validate our findings, we conducted ISG analysis on these datasets(GSE233277), revealing a significantly higher ISG score in INS patients than in healthy controls(Fig. S7A-S7D). Moreover, in addition to the activation of APRIL signaling mentioned by the authors, we found that BAFF signaling was activated in INS patients (Fig. S7E-S7F). Furthermore, we did not detect the expression of IFN-α or IFN-β. T cells have also been identified as the main contributors of IFN-γ(Fig. S7G). Collectively, the IFN-related pathways were affirmingly triggered during the disease.

### The efficacy of a BAFF inhibitor showed benefits in a case of INS patients

Based on the present findings, inhibitors targeting IFN, BAFF and its receptors may offer promising new strategies for INS patients.

Recently, an 11-year-old boy diagnosed with FRNS for eight years was admitted to our hospital. Adrenocorticotropic hormone(ACTH) and tacrolimus administration failed to maintain complete remission. To control frequent relapse, the patient was co-administered with telitacicept(bioagent targeting BAFF and APRIL, 80 mg per week, hypodermic injection) after achieving complete remission with corticosteroids. Remarkably, the patient remained free of relapse during nine months of follow-up(Fig. 6K). The long-term follow-up is ongoing. Furthermore, our center is conducting a clinical trial focusing on telitacicept in pediatric patients with FRNS or SDNS(STERN, NCT06125405). The benefits of telitacicept have also become apparent, suggesting a new option for clinical management. Moreover, belimumab(a monoclonal antibody targeting BAFF) demonstrated potential efficacy in pediatric FRNS by reducing the occurrence of relapse, notwithstanding the extremely small enrolled patients and limited follow-up duration [43]. Large samples of clinical trials and prolonged follow-ups are warranted.

## Discussion

Substantial research has consistently highlighted the immune-mediated nature of INS [44], whereas the exact mechanisms have not been elucidated. Our investigation revealed a distinctive subset of patient-specific T lymphocytes (IFI44L + CD4 + T cells) characterized by high expression of IFN-related genes. Activation of ISGs was also evident in distinct immune cells, as corroborated by qPCR. Furthermore, we employed scRNAseq, ELISA, and flow cytometry to ascertain the upregulation of IFN downstream pathways. Specifically, elevated expression of BAFF, triggered by IFN, along with its receptors BCMA and TACI in plasma cells, contributes to active disease states. Altogether, our study highlights the activation of IFN signaling in pediatric steroid-sensitive INS pathogenesis.

With clarification of IFN activation, our study highlighted that type II IFNs, play a predominant role in the source of INS, in accordance with the previous studies (the levels of IFN-γ increased in INS) [8, 45]. However, Stefanović V et al. [46] reported lower levels of IFN-γ in 11 relapsing INS patients compared to healthy controls, which may influenced by the limited sample size. In addition, prolonged clinical treatment with injections of recombinant IFN-α has been reported to induce NS in several diseases [11, 47, 48]. Levy DE suggested that IFN-γ enhances ISGF3 synthesis, strengthening the IFN-α response by approximately tenfold compared with IFN-α alone [49]. Further analysis is needed to determine whether IFN-γ, independently of or in association with IFN-α, impacts INS pathogenesis.

T and NK cells are the main contributors to IFN-γ [42]. In our study, T cells were identified as the essential contributors of IFN-γ. This finding aligns with Guimarães FTL et al. [50], who observed that IFN-γ expression on CD4 + T cells was more remarkable in INS patients than in controls. However, due to the limited sample size, the actual subcluster responsible for this signaling is still controversial, necessitating further large-scale and in-depth analyses. Overall, we have shown that the activation of IFN-γ, irrespective of its source, inhibiting IFN-γ or its downstream targets like JAK and STAT inhibitors, may offer benefits in INS.

Besides the upstream of IFN, the downstream pathways of IFN are also correlated with pathogenesis. Our study proposed that IFN-γ stimulates the release of BAFFs, interacting with receptors(TACI, BCMA) on B cells and activating autoantibody-producing cells. Our results align with previous studies that a significantly greater level of BAFF is a hallmark of untreated INS [38, 39]. In addition, BAFF and BCMA are considered potential biomarkers of other immune diseases, like SLE [51, 52]. Al-Aubodah TA et al. [40] suggested APRIL stimulates antibody production in NS. The effectiveness of belimumab and telitacicept, which target BAFF or are associated with APRIL, in treating SLE has been established [53, 54], and its efficacy in treating INS has also shown promising results. Further investigations are required to elucidate the role of BAFF, either independently or in conjunction with APRIL signaling, in the pathogenesis of INS.

Our study demonstrated that adaptive immune cells, particularly IFN-γ producing B cell maturation CD4 + T cells, drive B cell maturation and pathogenic autoantibody production in NS. Emerging evidence further implicates innate immune cells, such as basophils, in glomerular disease. Basophils have been implicated in B cell function and disease activity by mediating Th2 cytokines production(e.g., IL4, IL13) and modeling Th1/Th2 imbalance [55]. Additionally, the activation and recruitment of basophils facilitate autoantibodies production and promote glomerular deposition of circulating immune complexes, as reported in lupus nephritis, and can be have a broader role in disease characterized by a distinct IFN-I signature, with IFN-γ also contributing to the pathogenesis [12, 56, 57]. While our PBMC-focused scRNA-seq analysis did not solve rare basophil populations due to their low abundance, further studies should employ advanced single-cell analysis to clarify their role in INS pathogenesis.

Previous studies have indicated that glucocorticoids mitigate the activation of STAT1, which is mediated by TLR4 through suppressor of cytokine signaling 1 (SOCS1), thereby inhibiting IFN-related pathways [58]. Another study highlighted that glucocorticoid-induced suppression of T-cell activity results in diminishing response of TH1 and TH17 cells [59]. Despite these findings, the target of steroids in INS remains elusive. Our analysis revealed that the cumulative activation of IFN pathways was suppressed after steroid treatment, indicating that steroids may inhibit the IFN pathway. However, the specific mechanisms of steroids on pathology still require further investigation.

Despite these crucial findings, our study had several limitations. First, our study focused on new-onset steroid-sensitive NS patients, constraining the generalizability of findings to steroid-resistant, frequent relapsing, or congenital NS patients. Second, the sample size used for scRNA-seq was relatively small, which may have impacted our ability to identify significant changes in the proportion of subclusters accurately. The limited sample sizes for qPCR, flow cytometry, and ELISA may have affected the robustness of the verification. Additionally, Our analysis didn’t investigate the mechanistic role of innate immune cells(e.g., basophils) in INS pathogenesis and the absence of a well-established model for INS also hinders our ability to confirm the mechanism in depth in cellular and animal models. This limitation necessitates our reliance on patient PBMCs to demonstrate the participation of IFN. Although our study revealed some intriguing findings, a large sample size and a well-established model are essential for further understanding the role of IFN pathways in NS.

## Conclusion

Our study is noteworthy as it is the first to comprehensively describe all immune cells in PBMCs before and after steroid treatment in pediatric patients with new-onset steroid-sensitive INS. We substantiated the significance of IFN-related pathway activation in the pathogenesis of INS. This activation leads to the release of autoantibodies from autoantibody-producing cells, driven mainly by IFN-γ, through the interaction of BAFF with its receptors(TACI, BCMA) on B cells. This work also proposes potential therapeutic strategies such as inhibiting IFN itself and its associated pathway or suppressing the activation of BAFF and B-cell surface receptors. Further studies are required to address INS heterogeneity and its recurrence.

## Supplementary Information

Supplementary Material 1. Supplementary Fig. 1. Quality control and baseline data of each enrolled sample. (A). Principal component analysis before and after processing in Harmony. (B). t-SNE projections among different groups. (C). Quality control of the scRNA-seq data. (D). t-SNE projections from all enrolled samples. t-SNE in the control group (left), STS Pre group (middle), and STS Post group (right). (E). Boxplot comparing the proportion of plasmacytoid dendritic cells(pDCs) across the groups. The STS Pre vs. CT and STS Post vs. CT sample comparisons show exact P values determined by the Wilcoxon rank-sum test. Pre- vs. post-STS scores were calculated via the paired two-sample Wilcoxon signed-sum test. (F). The baseline information of patients and healthy controls enrolled in the scRNA-seq cohort. Supplementary Fig. 2. Focused analysis of T cells and pDCs. (A). UMAP embedding of T lymphocytes from all profiled samples in different groups. UMAP in the control group (left), STS Pre group (middle), and STS Post group (right). (B). Boxplot comparing the proportions of CRIP + CD4 + T cells, NK T cells, and TRGC2 + CD8 + T cells across the groups. The exact P values determined by the Wilcoxon rank-sum test are shown for the STS Pre vs. CT and STS Post vs. CT comparisons. Differences between STS Pre and STS Post were evaluated by the paired two-sample Wilcoxon signed-sum test. (C). Enriched pathways from Gene Ontology Biological Process Enrichment Analysis for TRGC + CD8 + T cells. (D). Enriched pathways identified by Gene Ontology Biological Process enrichment analysis in NEAT + T cells. (E). Heatmap representing the enrichment of MSigDB Hallmark gene sets for each T lymphocyte subtype across groups. (F). Pseudotime trajectory analysis of CD4 + T lymphocyte subtypes. (G). Heatmap represents DEGs within pDCs across groups. (H). Heatmap representing the enrichment of MSigDB Hallmark gene sets in the MSigDB of each group within pDCs. Supplementary Fig. 3. Focused analysis of B cells and myeloid cells. (A). Heatmap representing the enrichment of Hallmark gene sets in the MSigDB for each cell type within B lymphocytes across groups. (B). Boxplot comparing the proportions of myeloid cells across the groups. The exact P values determined by the Wilcoxon rank-sum test are shown for the STS Pre vs. CT and STS Post vs. CT comparisons. Differences between STS Pre and STS Post were evaluated by the paired two-sample Wilcoxon signed-sum test. (C). Enriched pathways from Gene Ontology Biological Process Enrichment Analysis for CD16 + monocytes. (D). Heatmap representing the enrichment of MSigDB Hallmark gene sets in each monocyte cell type across groups. Supplementary Fig. 4. The characteristics of IFN-related genes involved in pathogenesis. (A). Heatmap showing the differentially expressed genes (DEGs) in classical dendritic cells(cDCs) across groups. (B). Heatmap showing the genes differentially expressed in mast cells across groups. (C). Heatmap representing the enrichment of MSigDB Hallmark gene sets in the mast cells across groups. (D). Heatmap representing the enrichment of MSigDB Hallmark gene sets in the cDC across groups. (E). Heatmap representing the enrichment of Hallmark gene sets in the MSigDB for each cell type within Natural killer (NK) cells across groups. (F). Venn plot of the overlapping genes downregulated in the STS Pre group among B lymphocytes, T lymphocytes, monocytes, NK cells, cDCs and pDCs. (G). Receiver operating characteristic (ROC) curve and area under the curve (AUC) of overlapping genes expressed at lower levels before treatment in all cell types. (H). T-SNE analysis of CXCR4 expression in the three groups. (I). The relative expression of CXCR4 across groups was determined through qPCR. Statistical significance is denoted as P < 0.05 (*), P < 0.01 (**), P < 0.001 (***), or P < 0.0001 (****). Supplementary Fig. 5. Analysis of transcription factors (TFs) in INS. (A). Heatmap showing TFs activation across the subcluster of T lymphocytes. (B). Heatmap showing TFs activation across the subclusters of monocytes, cDCs, neutrophils and mast cells. (C). Heatmap showing TFs activation across the subclusters of B lymphocytes. (D). Heatmap showing TFs activation across the subcluster among ΝΚ cells. (E). Heatmap showing the expression level of IFNs (IFNA1, IFNA2, IFNB1, IFNG, IFNL1, IFNL2, and IFNL3). in each cell type across groups. Supplementary Fig. 6. (A). Surface expression data of TACI and BCMA on naïve B cells, unswitched memory B cells, switched memory B cells and plasma cells determined via flow cytometry. The samples were obtained from INS patients. (B). Surface expression data of TACI and BCMA on naïve B cells, unswitched memory B cells, switched memory B cells and plasma cells determined via flow cytometry. The samples were obtained from healthy donors. (C). The proportion of naïve B cells in the serum of NS patients compared to that of healthy individuals. ns, p > 0.05. (D). The proportion of unswitched memory B cells in the serum of NS patients compared to that of healthy individuals. ns, p > 0.05. (E). The proportion of switched memory B cells in the serum of NS patients compared to that in the serum of healthy individuals. ns, p > 0.05. (F). The proportion of plasma cells in the serum of NS patients compared to that in the serum of healthy individuals. ns, p > 0.05. Supplementary Fig. 7. Supplementary analysis from an extra INS cohort (GEO233277) also validates the activation of IFN. (A). UMAP dimensionality reduction embedding from GEO datasets. (B). Heatmap showing the expression levels of the markers across each cell type using scRNAseq from the GEO datasets. The color intensity indicates the marker of interest. (C). Violin plot of the ISGs across groups using scRNAseq from the GEO datasets. Significance was evaluated with the Wilcoxon rank-sum test. (D). ISG scores among cell subtypes across groups using scRNAseq from the GEO datasets. Significance was evaluated with the Wilcoxon rank-sum test. Statistical significance is denoted as P < 0.05 (*), P < 0.01 (**), P < 0.001 (***), or P < 0.0001 (****). (E). Incoming signaling patterns of APRIL and BAFF across groups using scRNAseq from the GEO datasets. (F). Outgoing signaling patterns of APRIL and BAFF across groups using scRNAseq from the GEO datasets. (G). Heatmap showing the expression level of all kinds of IFNs in each cell type across groups using scRNAseq from the GEO datasets. Supplementary Fig. 8. The potential mechanism underlying the pathogenesis of pediatric idiopathic nephrotic syndrome. (A). IFN-γ activation, mainly generated by T cells, stimulates the upregulation of BAFF expression among monocytes, dendritic cells, and neutrophils. Subsequently, the activation of BAFF interacts with its receptors on B cells, especially BCMA and TACI, facilitating B cell maturation and leading to autoantibody release. BAFF: B-cell activating factor; BCMA: B-cell maturation antigen; TACI: transmembrane activator and cyclophilin ligand interactor.

Supplementary Material 2. Supplementary Table 1. Inclusion and exclusion criteria of nephrotic syndrome patients. Supplementary Table 2. Detailed information of patients and healthy controls. Supplementary Table 3. Genes highly expressed in each cluster (T cells, B cells, NK cells, monocytes, cDC, pDC, and mast cells) in the STS Pre group. Supplementary Table 4. Genes lowly expressed in each cluster (T cells, B cells, NK cells, monocytes, cDC, pDC, and mast cells) in the STS Pre group. Supplementary Table 5. Enrichment of MSigDB Hallmark gene sets within each major cluster (T cells, B cells, NK cells, monocytes, cDC, pDC, and mast cells) across groups. Supplementary Table 6. Enrichment of MSigDB Hallmark gene sets within each subcluster of T cells, B cells, NK cells, and monocytes across groups. Supplementary Table 7. Interferon-stimulated genes. Supplementary Table 8. RSS scores across groups within each subcluster of T, B, NK, and myeloid cells.

## Data Availability

Data is provided in the  Availability of data and materials within the manuscript.The scRNA-seq data are publicly available in the Zenodo repository under the DOI: 10.5281/zenodo.13765197. Moreover, single cell RNA-sequencing data retrieved from the Gene Expression Omnibus (GEO) at  http://www.ncbi.nlm.nih.gov/geo under series accession number GSE214865 were used as the control group. Additionally, data from the series accession number GSE233277 were employed for complementary analysis.

## Abbreviations

PBMCs: Peripheral blood mononuclear cells
INS: Idiopathic nephrotic syndrome
qPCR: Quantitative polymerase chain reaction
ELISA: Enzyme-linked immunosorbent assay
ISGs: Interferon-stimulated genes
IFN: Interferon
BAFF: B-cell activating factor
FRNS: Frequent relapse NS
SD: Steroid dependency nephrotic syndrome
FSGS: Focal segmental glomerulosclerosis
RTX: Rituximab
IL: Interleukin
TACI: Transmembrane activator and cyclophilin ligand interactor
BCMA: B-cell maturation antigen
GEO: Gene Expression Omnibus
SNN: Shared nearest neighbor
UMAP: Uniform manifold approximation and projection
t-SNE: T-distributed stochastic neighbor embedding
DEG: Differentially expressed gene
GO: Gene Ontology
TFs: Transcription factors
NK cells: Natural killer
PDCs: Plasmacytoid dendritic cells
CTLs: Cytotoxic T lymphocytes
CMs: Classical monocytes
NCMs: Nonclassical monocytes
IMs: Intermediate monocytes
*IRF*: Interferon regulatory factor
STAT: Signal transducer and activator of transcription
ACTH: Adrenocorticotropic hormone
SOCS1: Suppressor of cytokine signaling 1
