# Supplementary figures and images for: Single cell analysis identified IFN signaling activation contributes to the pathogenesis of pediatric steroid-sensitive nephrotic syndrome
Source: Biomark Res. 2025 May 24;13:77. doi: 10.1186/s40364-025-00790-2 (PMC12103753; doi:10.1186/s40364-025-00790-2)

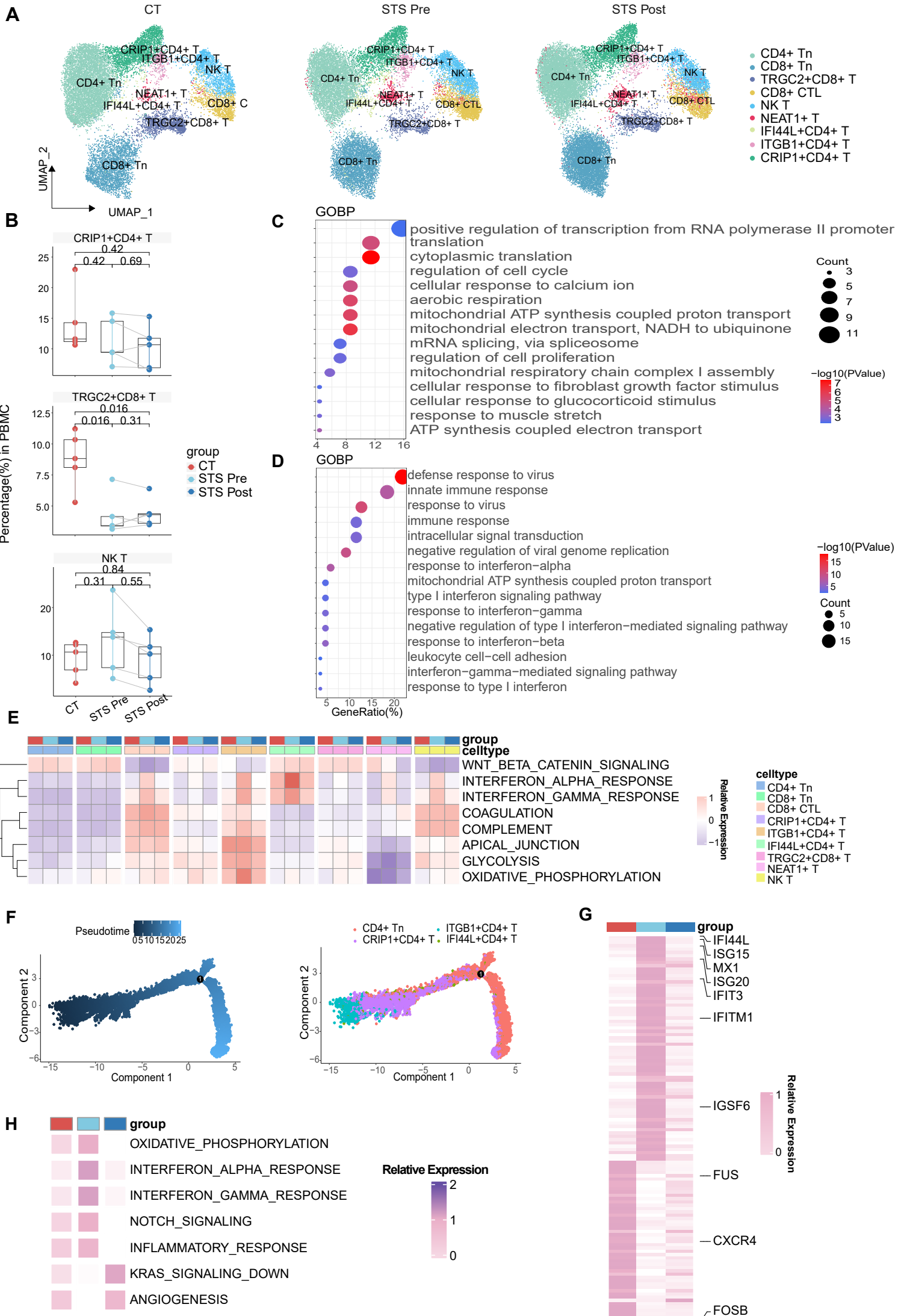

Supplement: Supplementary file 1 — Supplementary Material 1. Supplementary Fig. 1. Quality control and baseline data of each enrolled sample. (A). Principal component analysis before and after processing in Harmony. (B). t-SNE projections among different groups. (C). Quality control of the scRNA-seq data. (D). t-SNE projections from all enrolled samples. t-SNE in the control group (left), STS Pre group (middle), and STS Post group (right). (E). Boxplot comparing the proportion of plasmacytoid dendritic cells(pDCs) across the groups. The STS Pre vs. CT and STS Post vs. CT sample comparisons show exact P values determined by the Wilcoxon rank-sum test. Pre- vs. post-STS scores were calculated via the paired two-sample Wilcoxon signed-sum test. (F). The baseline information of patients and healthy controls enrolled in the scRNA-seq cohort. Supplementary Fig. 2. Focused analysis of T cells and pDCs. (A). UMAP embedding of T lymphocytes from all profiled samples in different groups. UMAP in the control group (left), STS Pre group (middle), and STS Post group (right). (B). Boxplot comparing the proportions of CRIP + CD4 + T cells, NK T cells, and TRGC2 + CD8 + T cells across the groups. The exact P values determined by the Wilcoxon rank-sum test are shown for the STS Pre vs. CT and STS Post vs. CT comparisons. Differences between STS Pre and STS Post were evaluated by the paired two-sample Wilcoxon signed-sum test. (C). Enriched pathways from Gene Ontology Biological Process Enrichment Analysis for TRGC + CD8 + T cells. (D). Enriched pathways identified by Gene Ontology Biological Process enrichment analysis in NEAT + T cells. (E). Heatmap representing the enrichment of MSigDB Hallmark gene sets for each T lymphocyte subtype across groups. (F). Pseudotime trajectory analysis of CD4 + T lymphocyte subtypes. (G). Heatmap represents DEGs within pDCs across groups. (H). Heatmap representing the enrichment of MSigDB Hallmark gene sets in the MSigDB of each group within pDCs. Supplementary Fig. 3. Focused analys [file 40364_2025_790_MOESM1_ESM.zip › Supplementary Figure 2-.pdf]

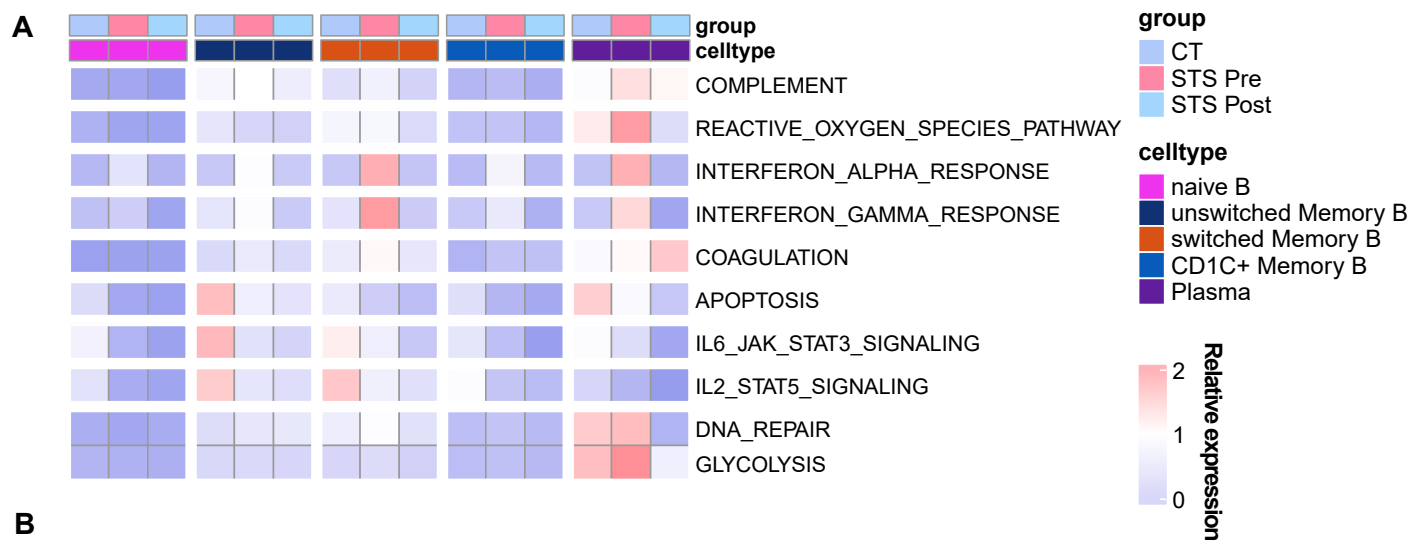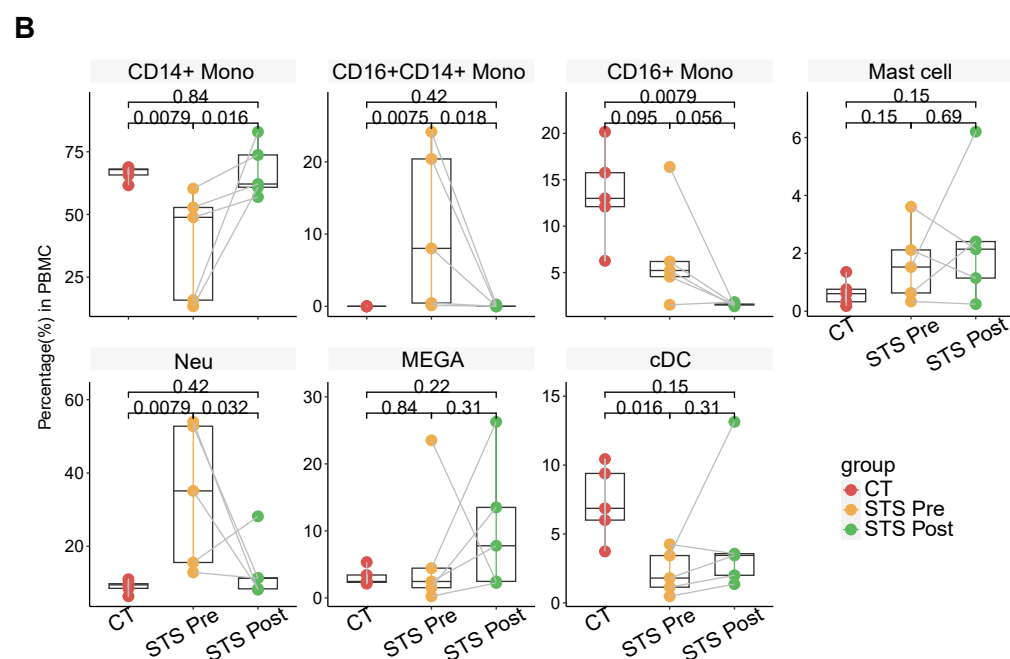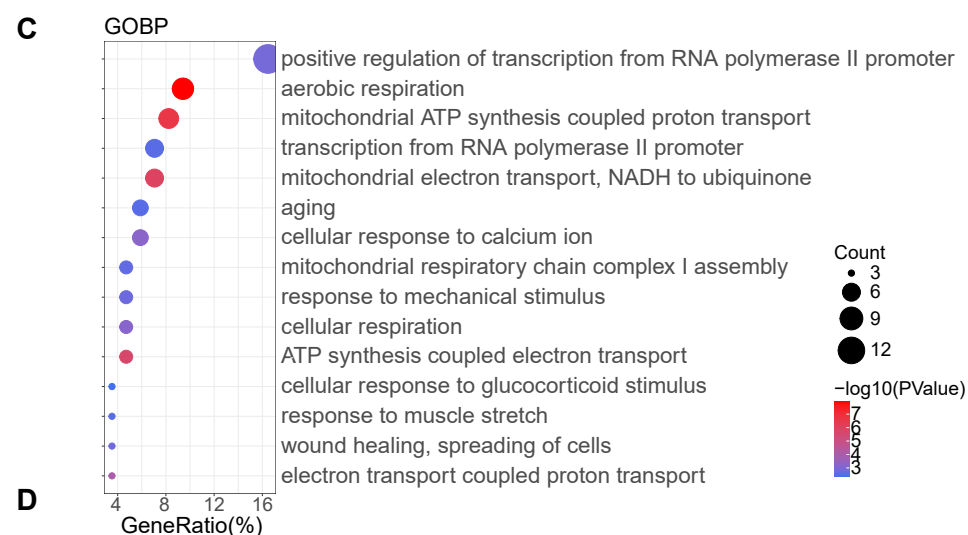

Supplement: Supplementary file 1 — Supplementary Material 1. Supplementary Fig. 1. Quality control and baseline data of each enrolled sample. (A). Principal component analysis before and after processing in Harmony. (B). t-SNE projections among different groups. (C). Quality control of the scRNA-seq data. (D). t-SNE projections from all enrolled samples. t-SNE in the control group (left), STS Pre group (middle), and STS Post group (right). (E). Boxplot comparing the proportion of plasmacytoid dendritic cells(pDCs) across the groups. The STS Pre vs. CT and STS Post vs. CT sample comparisons show exact P values determined by the Wilcoxon rank-sum test. Pre- vs. post-STS scores were calculated via the paired two-sample Wilcoxon signed-sum test. (F). The baseline information of patients and healthy controls enrolled in the scRNA-seq cohort. Supplementary Fig. 2. Focused analysis of T cells and pDCs. (A). UMAP embedding of T lymphocytes from all profiled samples in different groups. UMAP in the control group (left), STS Pre group (middle), and STS Post group (right). (B). Boxplot comparing the proportions of CRIP + CD4 + T cells, NK T cells, and TRGC2 + CD8 + T cells across the groups. The exact P values determined by the Wilcoxon rank-sum test are shown for the STS Pre vs. CT and STS Post vs. CT comparisons. Differences between STS Pre and STS Post were evaluated by the paired two-sample Wilcoxon signed-sum test. (C). Enriched pathways from Gene Ontology Biological Process Enrichment Analysis for TRGC + CD8 + T cells. (D). Enriched pathways identified by Gene Ontology Biological Process enrichment analysis in NEAT + T cells. (E). Heatmap representing the enrichment of MSigDB Hallmark gene sets for each T lymphocyte subtype across groups. (F). Pseudotime trajectory analysis of CD4 + T lymphocyte subtypes. (G). Heatmap represents DEGs within pDCs across groups. (H). Heatmap representing the enrichment of MSigDB Hallmark gene sets in the MSigDB of each group within pDCs. Supplementary Fig. 3. Focused analys [file 40364_2025_790_MOESM1_ESM.zip › supplementary figure 3.pdf]

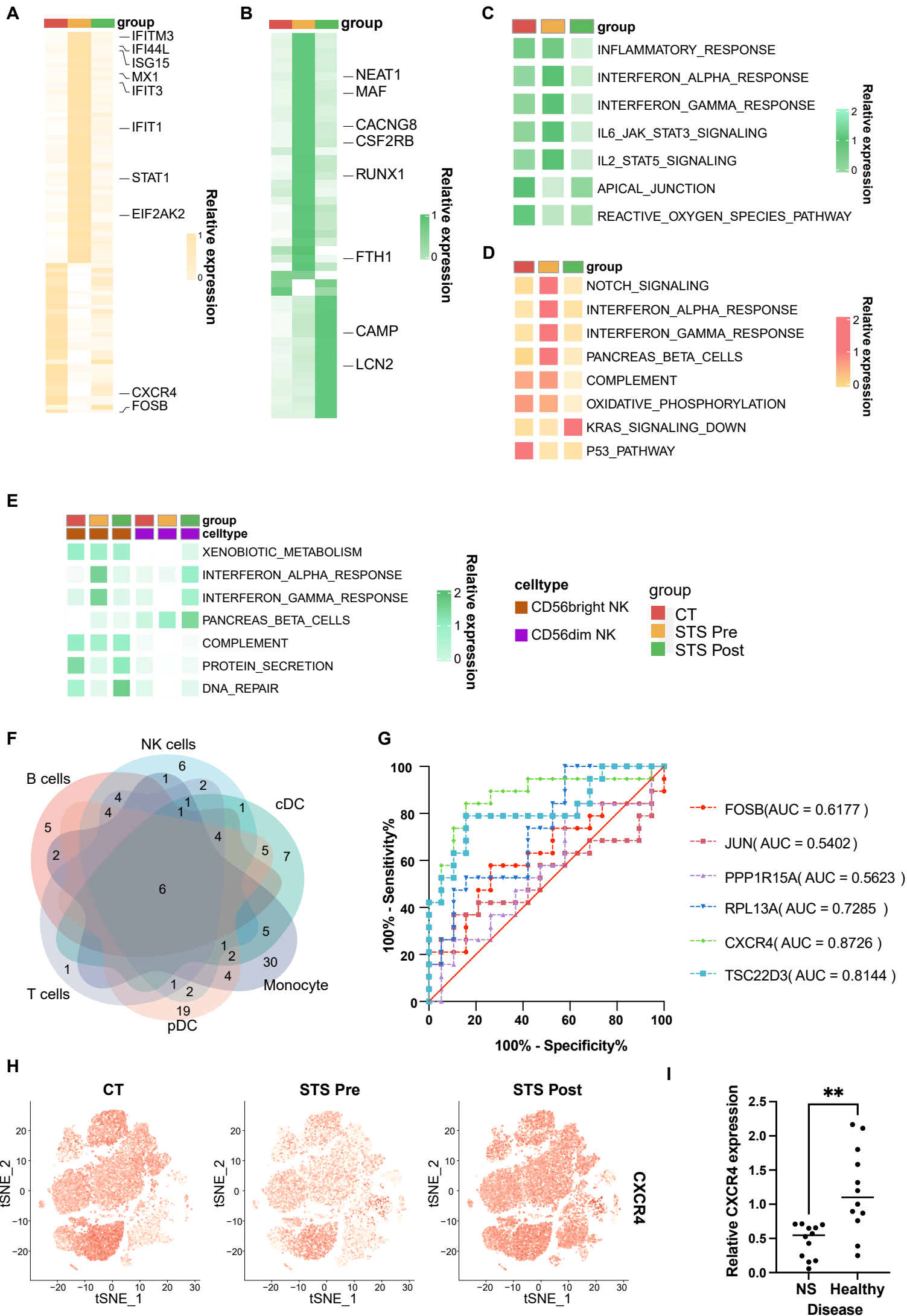

Supplement: Supplementary file 1 — Supplementary Material 1. Supplementary Fig. 1. Quality control and baseline data of each enrolled sample. (A). Principal component analysis before and after processing in Harmony. (B). t-SNE projections among different groups. (C). Quality control of the scRNA-seq data. (D). t-SNE projections from all enrolled samples. t-SNE in the control group (left), STS Pre group (middle), and STS Post group (right). (E). Boxplot comparing the proportion of plasmacytoid dendritic cells(pDCs) across the groups. The STS Pre vs. CT and STS Post vs. CT sample comparisons show exact P values determined by the Wilcoxon rank-sum test. Pre- vs. post-STS scores were calculated via the paired two-sample Wilcoxon signed-sum test. (F). The baseline information of patients and healthy controls enrolled in the scRNA-seq cohort. Supplementary Fig. 2. Focused analysis of T cells and pDCs. (A). UMAP embedding of T lymphocytes from all profiled samples in different groups. UMAP in the control group (left), STS Pre group (middle), and STS Post group (right). (B). Boxplot comparing the proportions of CRIP + CD4 + T cells, NK T cells, and TRGC2 + CD8 + T cells across the groups. The exact P values determined by the Wilcoxon rank-sum test are shown for the STS Pre vs. CT and STS Post vs. CT comparisons. Differences between STS Pre and STS Post were evaluated by the paired two-sample Wilcoxon signed-sum test. (C). Enriched pathways from Gene Ontology Biological Process Enrichment Analysis for TRGC + CD8 + T cells. (D). Enriched pathways identified by Gene Ontology Biological Process enrichment analysis in NEAT + T cells. (E). Heatmap representing the enrichment of MSigDB Hallmark gene sets for each T lymphocyte subtype across groups. (F). Pseudotime trajectory analysis of CD4 + T lymphocyte subtypes. (G). Heatmap represents DEGs within pDCs across groups. (H). Heatmap representing the enrichment of MSigDB Hallmark gene sets in the MSigDB of each group within pDCs. Supplementary Fig. 3. Focused analys [file 40364_2025_790_MOESM1_ESM.zip › Supplementary Figure 4.pdf]

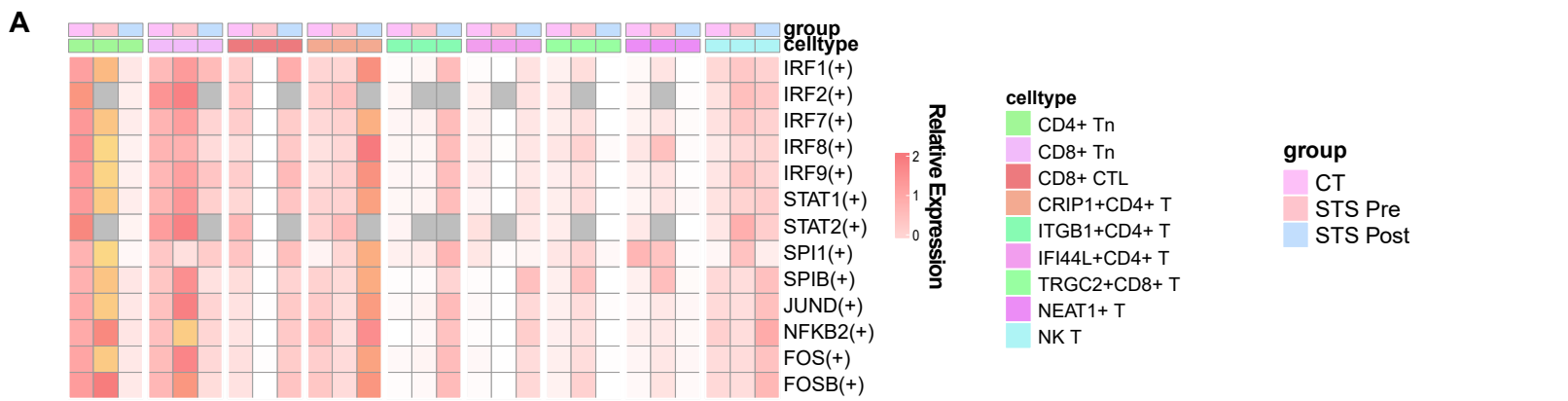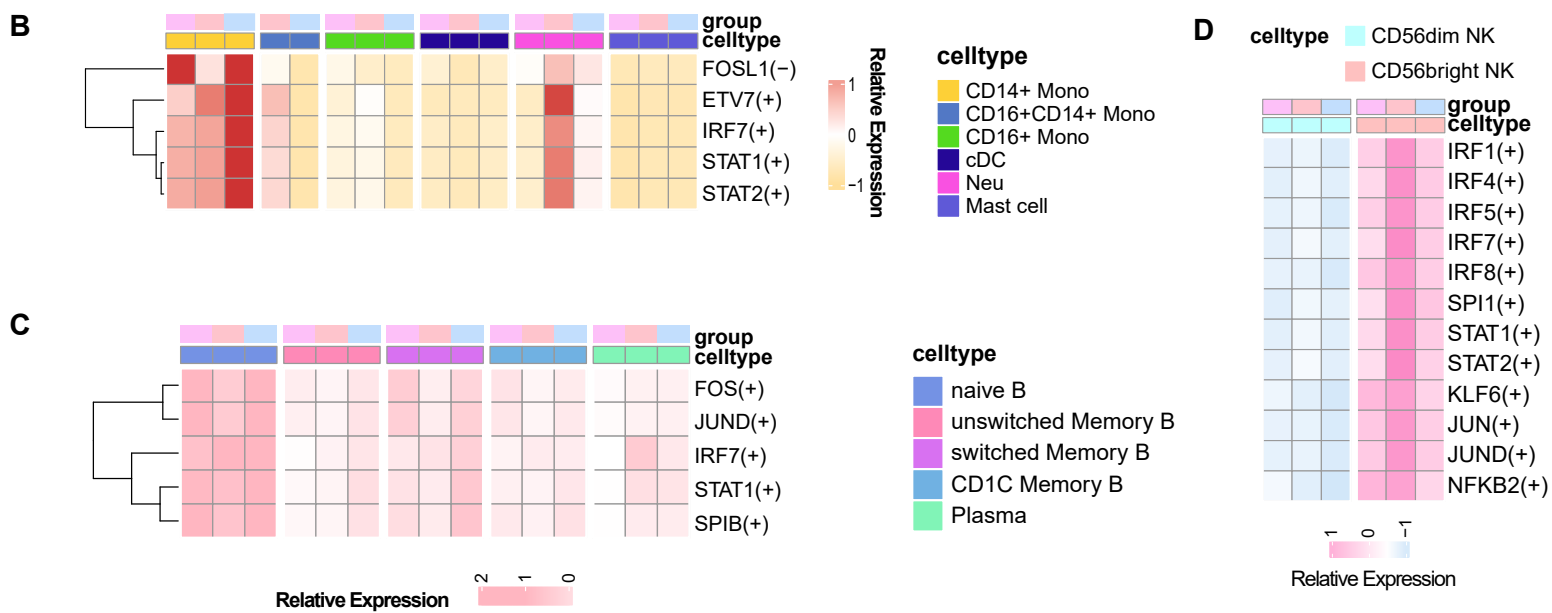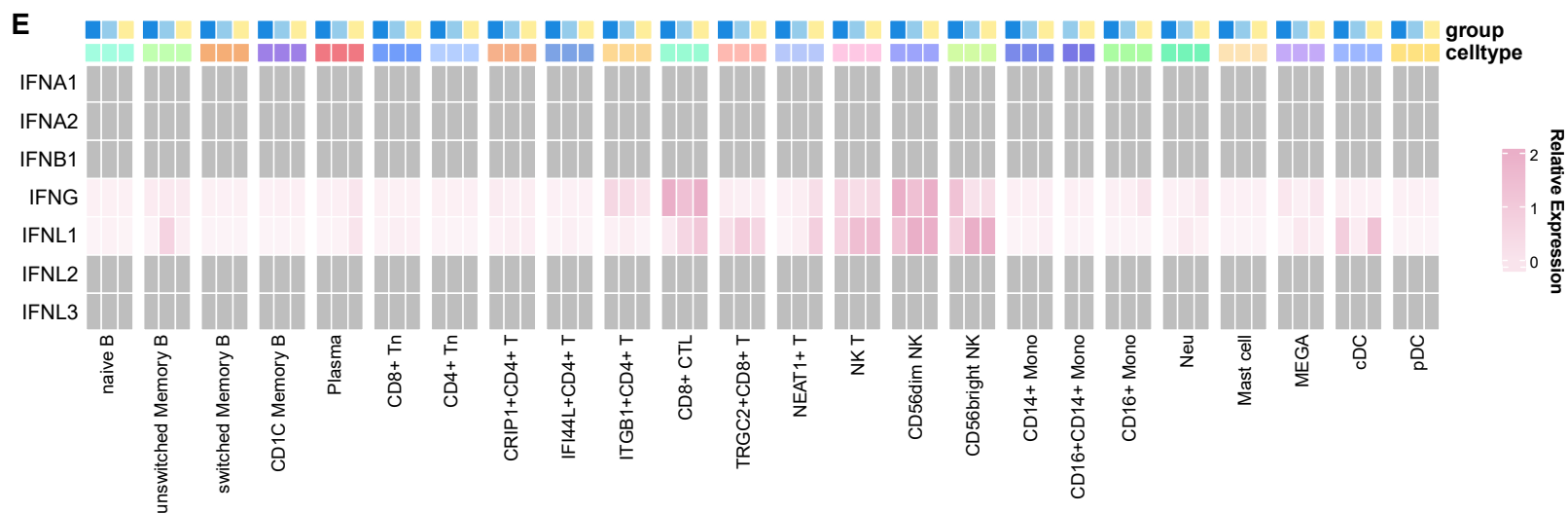

Supplement: Supplementary file 1 — Supplementary Material 1. Supplementary Fig. 1. Quality control and baseline data of each enrolled sample. (A). Principal component analysis before and after processing in Harmony. (B). t-SNE projections among different groups. (C). Quality control of the scRNA-seq data. (D). t-SNE projections from all enrolled samples. t-SNE in the control group (left), STS Pre group (middle), and STS Post group (right). (E). Boxplot comparing the proportion of plasmacytoid dendritic cells(pDCs) across the groups. The STS Pre vs. CT and STS Post vs. CT sample comparisons show exact P values determined by the Wilcoxon rank-sum test. Pre- vs. post-STS scores were calculated via the paired two-sample Wilcoxon signed-sum test. (F). The baseline information of patients and healthy controls enrolled in the scRNA-seq cohort. Supplementary Fig. 2. Focused analysis of T cells and pDCs. (A). UMAP embedding of T lymphocytes from all profiled samples in different groups. UMAP in the control group (left), STS Pre group (middle), and STS Post group (right). (B). Boxplot comparing the proportions of CRIP + CD4 + T cells, NK T cells, and TRGC2 + CD8 + T cells across the groups. The exact P values determined by the Wilcoxon rank-sum test are shown for the STS Pre vs. CT and STS Post vs. CT comparisons. Differences between STS Pre and STS Post were evaluated by the paired two-sample Wilcoxon signed-sum test. (C). Enriched pathways from Gene Ontology Biological Process Enrichment Analysis for TRGC + CD8 + T cells. (D). Enriched pathways identified by Gene Ontology Biological Process enrichment analysis in NEAT + T cells. (E). Heatmap representing the enrichment of MSigDB Hallmark gene sets for each T lymphocyte subtype across groups. (F). Pseudotime trajectory analysis of CD4 + T lymphocyte subtypes. (G). Heatmap represents DEGs within pDCs across groups. (H). Heatmap representing the enrichment of MSigDB Hallmark gene sets in the MSigDB of each group within pDCs. Supplementary Fig. 3. Focused analys [file 40364_2025_790_MOESM1_ESM.zip › Supplementary Figure 5.pdf]

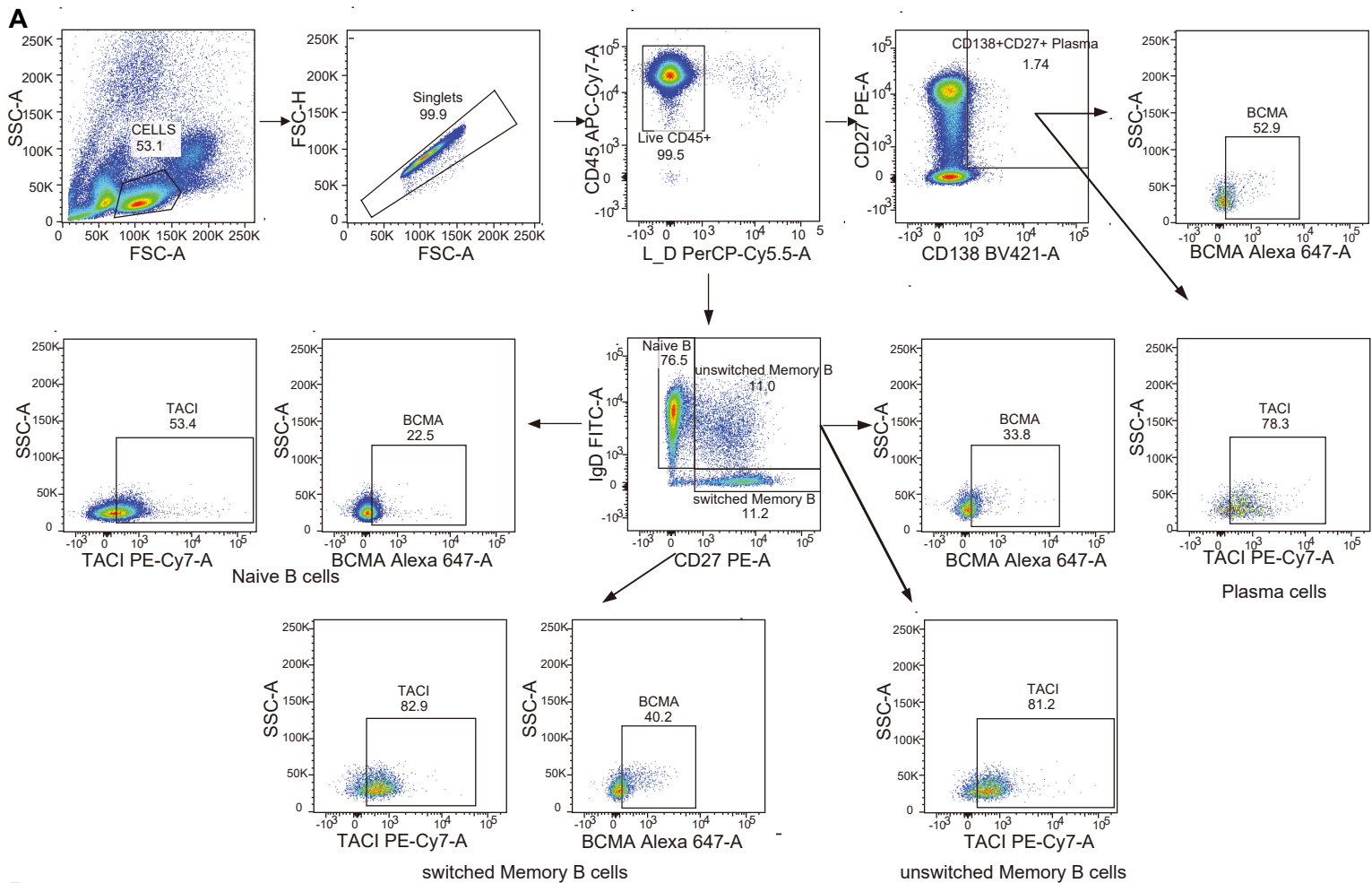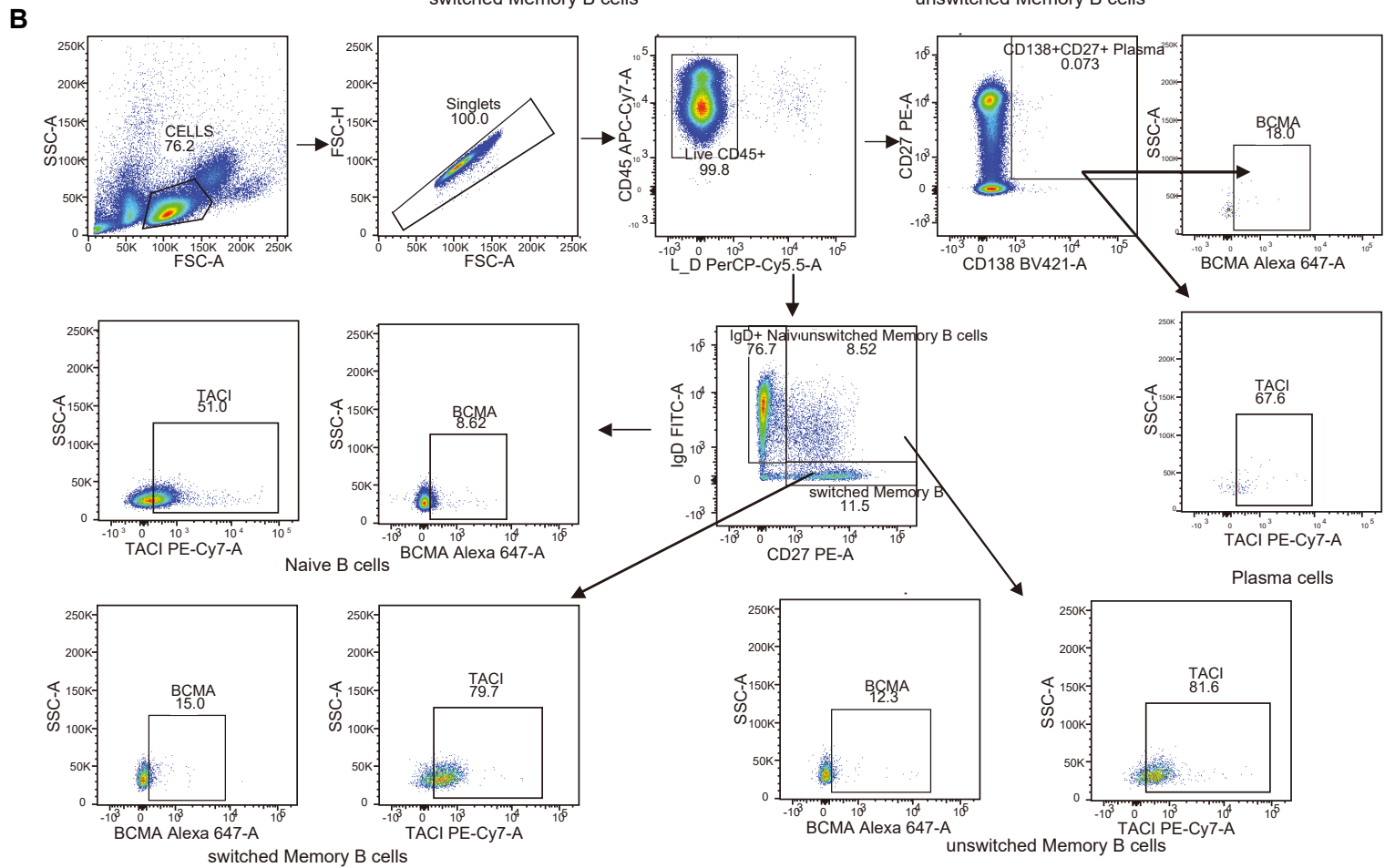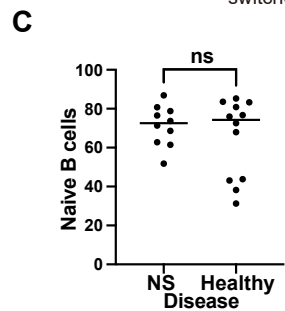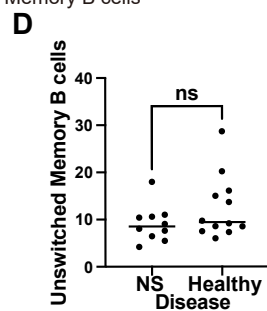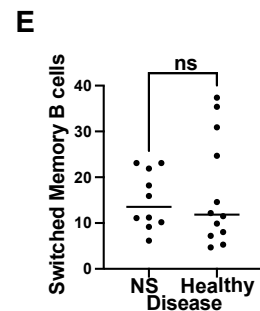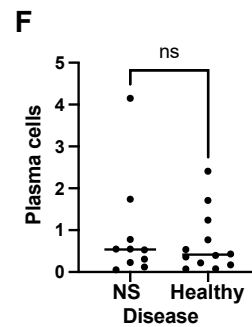

Supplement: Supplementary file 1 — Supplementary Material 1. Supplementary Fig. 1. Quality control and baseline data of each enrolled sample. (A). Principal component analysis before and after processing in Harmony. (B). t-SNE projections among different groups. (C). Quality control of the scRNA-seq data. (D). t-SNE projections from all enrolled samples. t-SNE in the control group (left), STS Pre group (middle), and STS Post group (right). (E). Boxplot comparing the proportion of plasmacytoid dendritic cells(pDCs) across the groups. The STS Pre vs. CT and STS Post vs. CT sample comparisons show exact P values determined by the Wilcoxon rank-sum test. Pre- vs. post-STS scores were calculated via the paired two-sample Wilcoxon signed-sum test. (F). The baseline information of patients and healthy controls enrolled in the scRNA-seq cohort. Supplementary Fig. 2. Focused analysis of T cells and pDCs. (A). UMAP embedding of T lymphocytes from all profiled samples in different groups. UMAP in the control group (left), STS Pre group (middle), and STS Post group (right). (B). Boxplot comparing the proportions of CRIP + CD4 + T cells, NK T cells, and TRGC2 + CD8 + T cells across the groups. The exact P values determined by the Wilcoxon rank-sum test are shown for the STS Pre vs. CT and STS Post vs. CT comparisons. Differences between STS Pre and STS Post were evaluated by the paired two-sample Wilcoxon signed-sum test. (C). Enriched pathways from Gene Ontology Biological Process Enrichment Analysis for TRGC + CD8 + T cells. (D). Enriched pathways identified by Gene Ontology Biological Process enrichment analysis in NEAT + T cells. (E). Heatmap representing the enrichment of MSigDB Hallmark gene sets for each T lymphocyte subtype across groups. (F). Pseudotime trajectory analysis of CD4 + T lymphocyte subtypes. (G). Heatmap represents DEGs within pDCs across groups. (H). Heatmap representing the enrichment of MSigDB Hallmark gene sets in the MSigDB of each group within pDCs. Supplementary Fig. 3. Focused analys [file 40364_2025_790_MOESM1_ESM.zip › Supplementary Figure 6.pdf]

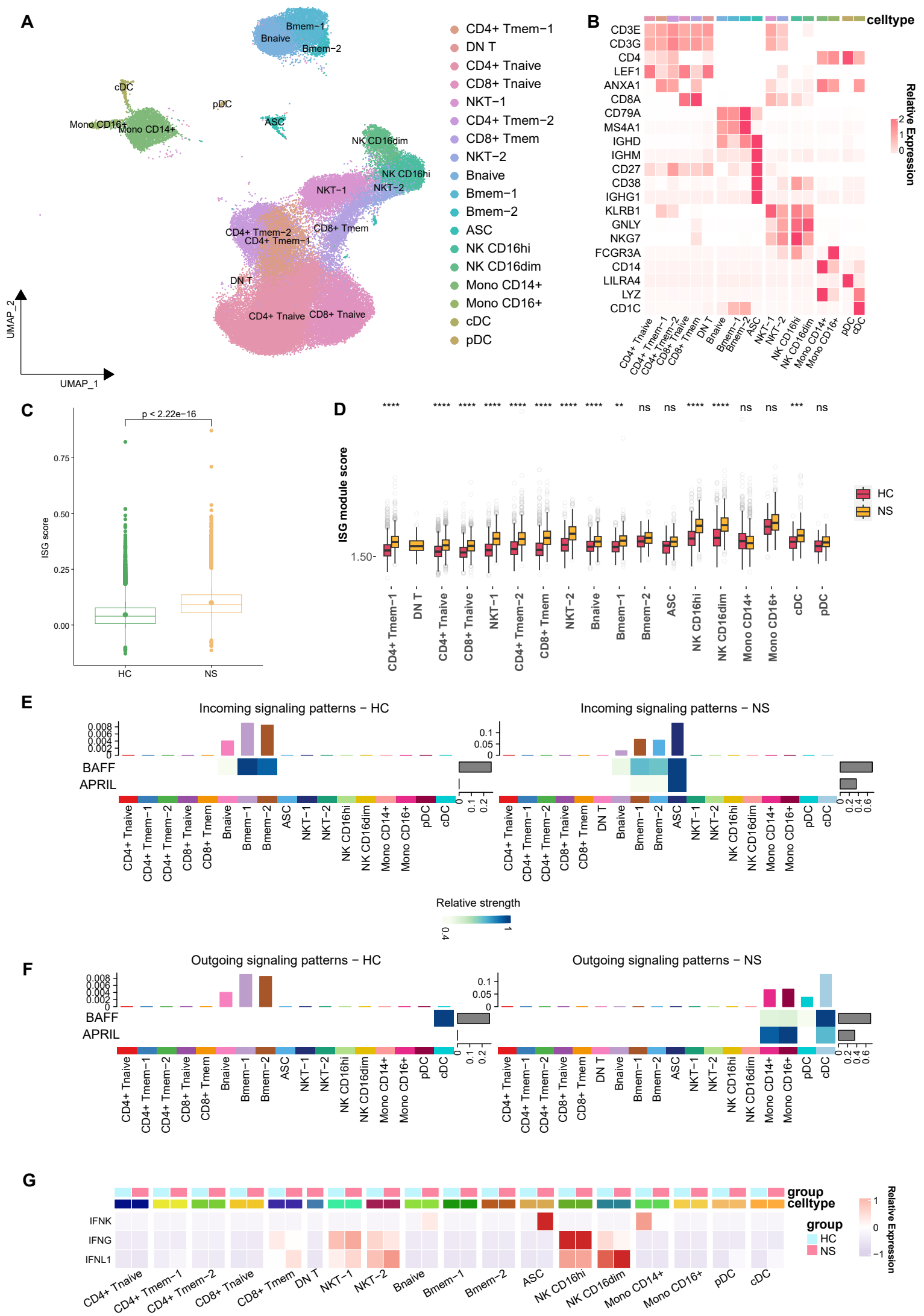

Supplement: Supplementary file 1 — Supplementary Material 1. Supplementary Fig. 1. Quality control and baseline data of each enrolled sample. (A). Principal component analysis before and after processing in Harmony. (B). t-SNE projections among different groups. (C). Quality control of the scRNA-seq data. (D). t-SNE projections from all enrolled samples. t-SNE in the control group (left), STS Pre group (middle), and STS Post group (right). (E). Boxplot comparing the proportion of plasmacytoid dendritic cells(pDCs) across the groups. The STS Pre vs. CT and STS Post vs. CT sample comparisons show exact P values determined by the Wilcoxon rank-sum test. Pre- vs. post-STS scores were calculated via the paired two-sample Wilcoxon signed-sum test. (F). The baseline information of patients and healthy controls enrolled in the scRNA-seq cohort. Supplementary Fig. 2. Focused analysis of T cells and pDCs. (A). UMAP embedding of T lymphocytes from all profiled samples in different groups. UMAP in the control group (left), STS Pre group (middle), and STS Post group (right). (B). Boxplot comparing the proportions of CRIP + CD4 + T cells, NK T cells, and TRGC2 + CD8 + T cells across the groups. The exact P values determined by the Wilcoxon rank-sum test are shown for the STS Pre vs. CT and STS Post vs. CT comparisons. Differences between STS Pre and STS Post were evaluated by the paired two-sample Wilcoxon signed-sum test. (C). Enriched pathways from Gene Ontology Biological Process Enrichment Analysis for TRGC + CD8 + T cells. (D). Enriched pathways identified by Gene Ontology Biological Process enrichment analysis in NEAT + T cells. (E). Heatmap representing the enrichment of MSigDB Hallmark gene sets for each T lymphocyte subtype across groups. (F). Pseudotime trajectory analysis of CD4 + T lymphocyte subtypes. (G). Heatmap represents DEGs within pDCs across groups. (H). Heatmap representing the enrichment of MSigDB Hallmark gene sets in the MSigDB of each group within pDCs. Supplementary Fig. 3. Focused analys [file 40364_2025_790_MOESM1_ESM.zip › Supplementary Figure 7-.pdf]

**A**

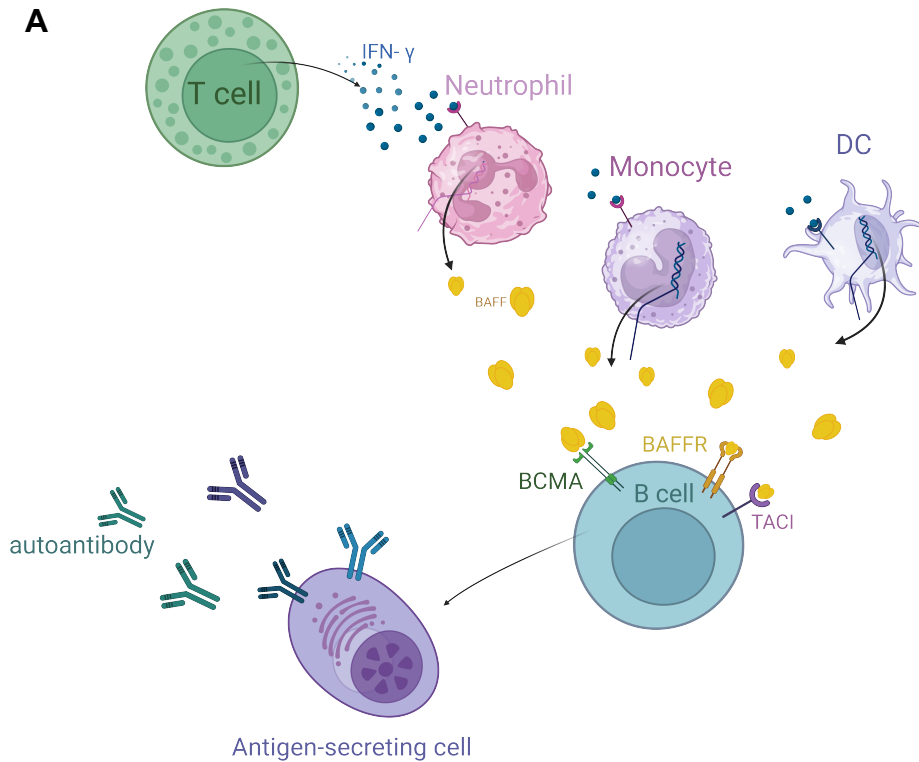

Supplement: Supplementary file 1 — Supplementary Material 1. Supplementary Fig. 1. Quality control and baseline data of each enrolled sample. (A). Principal component analysis before and after processing in Harmony. (B). t-SNE projections among different groups. (C). Quality control of the scRNA-seq data. (D). t-SNE projections from all enrolled samples. t-SNE in the control group (left), STS Pre group (middle), and STS Post group (right). (E). Boxplot comparing the proportion of plasmacytoid dendritic cells(pDCs) across the groups. The STS Pre vs. CT and STS Post vs. CT sample comparisons show exact P values determined by the Wilcoxon rank-sum test. Pre- vs. post-STS scores were calculated via the paired two-sample Wilcoxon signed-sum test. (F). The baseline information of patients and healthy controls enrolled in the scRNA-seq cohort. Supplementary Fig. 2. Focused analysis of T cells and pDCs. (A). UMAP embedding of T lymphocytes from all profiled samples in different groups. UMAP in the control group (left), STS Pre group (middle), and STS Post group (right). (B). Boxplot comparing the proportions of CRIP + CD4 + T cells, NK T cells, and TRGC2 + CD8 + T cells across the groups. The exact P values determined by the Wilcoxon rank-sum test are shown for the STS Pre vs. CT and STS Post vs. CT comparisons. Differences between STS Pre and STS Post were evaluated by the paired two-sample Wilcoxon signed-sum test. (C). Enriched pathways from Gene Ontology Biological Process Enrichment Analysis for TRGC + CD8 + T cells. (D). Enriched pathways identified by Gene Ontology Biological Process enrichment analysis in NEAT + T cells. (E). Heatmap representing the enrichment of MSigDB Hallmark gene sets for each T lymphocyte subtype across groups. (F). Pseudotime trajectory analysis of CD4 + T lymphocyte subtypes. (G). Heatmap represents DEGs within pDCs across groups. (H). Heatmap representing the enrichment of MSigDB Hallmark gene sets in the MSigDB of each group within pDCs. Supplementary Fig. 3. Focused analys [file 40364_2025_790_MOESM1_ESM.zip › Supplementary Figure 8.pdf]
